# Exploring the potential role of four *Rhizophagus irregularis* nuclear effectors: opportunities and technical limitations

**DOI:** 10.3389/fpls.2024.1384496

**Published:** 2024-04-24

**Authors:** María Victoria Aparicio Chacón, Sofía Hernández Luelmo, Viktor Devlieghere, Louis Robichez, Toon Leroy, Naomi Stuer, Annick De Keyser, Evi Ceulemans, Alain Goossens, Sofie Goormachtig, Judith Van Dingenen

**Affiliations:** ^1^ Department of Plant Biotechnology and Bioinformatics, Ghent University, Ghent, Belgium; ^2^ Center for Plant Systems Biology, VIB, Gent, Belgium

**Keywords:** arbuscular mycorrhizal symbiosis, effectors, mycorrhization, nuclear effector proteins, plant growth, protein-protein interaction, *Rhizophagus irregularis*, transcriptional responses

## Abstract

Arbuscular mycorrhizal fungi (AMF) are obligate symbionts that interact with the roots of most land plants. The genome of the AMF model species *Rhizophagus irregularis* contains hundreds of predicted small effector proteins that are secreted extracellularly but also into the plant cells to suppress plant immunity and modify plant physiology to establish a niche for growth. Here, we investigated the role of four nuclear-localized putative effectors, *i.e.*, GLOIN707, GLOIN781, GLOIN261, and RiSP749, in mycorrhization and plant growth. We initially intended to execute the functional studies in *Solanum lycopersicum*, a host plant of economic interest not previously used for AMF effector biology, but extended our studies to the model host *Medicago truncatula* as well as the non-host *Arabidopsis thaliana* because of the technical advantages of working with these models. Furthermore, for three effectors, the implementation of reverse genetic tools, yeast two-hybrid screening and whole-genome transcriptome analysis revealed potential host plant nuclear targets and the downstream triggered transcriptional responses. We identified and validated a host protein interactors participating in mycorrhization in the host.*S. lycopersicum* and demonstrated by transcriptomics the effectors possible involvement in different molecular processes, *i.e.*, the regulation of DNA replication, methylglyoxal detoxification, and RNA splicing. We conclude that *R. irregularis* nuclear-localized effector proteins may act on different pathways to modulate symbiosis and plant physiology and discuss the pros and cons of the tools used.

## 1 Introduction

Plants are sessile organisms that are exposed to various biotic and abiotic stresses against which they have developed sophisticated defense mechanisms (He et al., 2018; Gull et al., 2019). Biotic invaders, such as microbial pathogens, must overcome the multilayered plant immune system and change the plant’s physiology to successfully colonize the plant’s tissues and exploit the plant’s nutritional resources (Jones and Dangl, 2006; Dodds and Rathjen, 2010). To this end, pathogens often secrete so-called effector proteins that act on the outside or inside of plant cells (Lo Presti et al., 2015). Intracellularly, they interfere with numerous plant molecular pathways by binding host plant macromolecules or through the alteration of their biological activity (Lo Presti et al., 2015). This interplay occurs in different subcellular compartments, among which the nucleus, where effectors induce transcriptional reprogramming by binding the promoter region of specific plant genes or participate in posttranscriptional processing of specific mRNAs (Fu et al., 2007; Canonne and Rivas, 2012; Kim et al., 2020).

Additionally, plant symbionts use effectors to modulate host plant defense mechanisms and physiology. The arbuscular mycorrhizal (AM) symbiosis is one of the most well-characterized mutualistic relationships between roots of a wide range of land plants and AM fungi (AMF) belonging mainly to the Glomeromycotina subphylum (Parniske, 2008; Spatafora et al., 2016). Under phosphate-limiting conditions, plant roots accommodate the fungus that forms highly branched hyphal structures, the so-called arbuscules, inside plant cortical cells, in which the two partners exchange nutrients (Pimprikar and Gutjahr, 2018). Plants benefit from the fungal delivery of water and inorganic nutrients, mainly phosphorus, and, in return, favor fungal growth by transferring sugars and lipids (Jiang et al., 2017; Lanfranco et al., 2018). The genome of the AMF model species *Rhizophagus irregularis* encodes approximately 300 putative *in silico* predicted secreted effector proteins, implying their potential importance in symbiosis establishment and maintenance (Lin et al., 2014; Sędzielewska Toro and Brachmann, 2016; Kamel et al., 2017; Maeda et al., 2018; Zeng et al., 2018). However, to date, merely five of the predicted effector proteins, *i.e.*, SP7, SIS1, RiCRN1, RiSLM, and RiNLE1, have been functionally characterized.

Understanding the role of these effectors is a challenging task because the fungus is recalcitrant to efficient genetic modification, making it difficult to individuate the genetic evidence of function. Hence, reverse genetic tools are often the only way to address the problem. For the effector proteins mentioned above, a role in mycorrhization has been demonstrated by means of *Medicago truncatula* (barrel medic) composite plants with transgenic roots overexpressing and/or silencing the effector proteins (Kloppholz et al., 2011; Tsuzuki et al., 2016; Voß et al., 2018; Zeng et al., 2020; Wang et al., 2021). Of these five characterized *R. irregularis* effector proteins, three are nucleus compartmentalized. The targeted plant proteins and subsequent host pathways have only been identified for SP7 and RiNLE1, which possibly modulate *M. truncatula* host defense responses by two different strategies. SP7 interacts with the pathogenesis-related ETHYLENE RESPONSE FACTOR19 (ERF19) in the plant nucleus, where it regulates the expression of plant defense genes to boost AMF accommodation (Kloppholz et al., 2011), whereas RiNLE1 relies on the epigenetic regulation of *HISTONE 2B* (*H2B*), altering the expression of several host genes involved in immunity (Wang et al., 2021). Thus, although nearly 1/4^th^ of the putative *R. irregularis* effectors has a predicted nuclear localization (Zeng et al., 2018; Aparicio Chacón et al., 2023), the interacting host plant nuclear proteins and the triggered downstream transcriptional responses responsible for the plant host performance are largely unknown.

Here, we investigated four putative nuclear-localized effector proteins from *R. irregularis*, *i.e.*, GLOIN707, GLOIN781, GLOIN261, and RiSP749, using *Solanum lycopersicum* (tomato), a host plant of economic interest not previously used for AMF effector biology, *M. truncatula*, a well-known host and the non-host *Arabidopsis thaliana* (Arabidopsis) We found that they are potentially secreted by the fungus, localized in the plant host nucleus, and expressed during symbiosis in tomato. We identified their influence on plant growth and mycorrhization by ectopic expression of the effectors in Arabidopsis and *M. truncatula*, respectively. To gain insight into their molecular mode of action during mycorrhization, we examined which tomato nuclear plant proteins are targeted and which downstream transcriptional responses are triggered. Finally, we discuss the potential roles of these *R. irregularis* nuclear-localized effector proteins during AM symbiosis and consider the pros and cons of the tools used.

## 2 Materials and methods

### 2.1 Bioinformatic analysis

To find effector proteins, we mapped the 338 effectors identified by (Zeng et al., 2018) with the effectome database of the *R. irregularis* DAOM 197198 genome (taxid:747089) (Sędzielewska Toro and Brachmann, 2016; Kamel et al., 2017). Based on the putative nuclear localization sites (NLS) predicted by LOCALIZER 1.0.4 (Sperschneider et al., 2017), we started with 87 putative NLS-containing effector proteins. Of these 87, 54 were predicted to localize intracellularly (exclusion from the apoplast) by ApoplastP 1.0.1 (Sperschneider et al., 2018) and had a predicted signal peptide (SP) by SignalP v4.1 (Petersen et al., 2011) and SignalP v5.0 (Almagro Armenteros et al., 2019). From this list, we retained those that do not had a role in cell wall degradation and dismissed those that were predicted to be an integral part of the cell membrane with GO analysis, ending up with 41 candidates. We analyzed the homology at the amino acid identity level among these 41 effector candidates and retained those candidates that were not similar to mitigate potential redundancy in effector functions leading to a final list of 34 putative nuclear localized, secreted, non-apoplastic effectors. We then investigated their expression at 2, 4, and 6 weeks in mycorrhized tomato roots in a screening qRT-PCR analysis, resulting in the final selection of 10 effectors with interesting mycorrhiza-specific patterns (i.e. higher expression at 4 and/or 6 weeks post inoculation). From these, we could retrieve cDNA clones for five effectors from which four are represented here: GLOIN707 (GLOIN_2v1591707; RirG040740; GBC29935.2); GLOIN781 (GLOIN_2v1603781; jgi.p|Gloin1|349745; GBC42057.1); GLOIN261 (GLOIN_2v1478261; RirG045970; GBC25372.1) and RiSP749 (Gloin_2v1708442; RirG117440; GBC20232.1) (**Supplementary Figure 1**; **Supplementary Table 1**). Coding sequences (CDSs) were cloned and the resulting encoded effector proteins were aligned against the latest *R. irregularis* proteome annotation RIR17 using BlastP (https://nekko.nibb.ac.jp/blast/blast.html) (Maeda et al., 2018). To ascertain the conservation of *R. irregularis* effector-like proteins GLOIN707, GLOIN781, and GLOIN261 in other organisms, the full effector protein sequences were subjected to BlastP against nonredundant protein databases in NCBI and homologous effector candidates were selected following the criteria described by Wang et al. (2021) (with SignalP v5.0, effectorP 3.0 (Sperschneider and Dodds, 2022), and LOCALIZER software tools). Homologous nuclear effector-like protein candidates with SP were aligned and pairwise compared based on amino acid (AA) identities (**Supplementary Figure 2A**). Next, the phylogenetic relationship among nuclear effector-like homologous proteins was inferred by applying the Maximum Likelihood method with 1000 bootstrap replicates using CLC Workbench 8.1 software. The online tools HMMER v2.41.2 (https://www.ebi.ac.uk/Tools/hmmer/) and Conserved Domain Database (NCBI) were used to identify known functional domains from reference proteomes (**Supplementary Figure 2B**). For RiSP749, homologous proteins were also selected based on BlastP search against nonredundant protein databases in NCBI and only those proteins with an >60% identity were retained, resulting in 31 candidate homologous proteins from which only six were predicted to have an SP with SignalP v4.1 and 29 to have an NLS. All 29 nuclear homologous candidates were predicted to be cytoplasmic effectors with effectorP 3.0, although not all containing an SP. These 29 nuclear effector-like protein candidates were aligned and pairwise compared based on amino acid (AA) identities (**Supplementary Figure 2A**). Next, the phylogenetic relationship among these 29 candidates was inferred applying the Maximum Likelihood method with 1000 bootstrap replicates using CLC Workbench 8.1 software (**Supplementary Figure 2B**). The schematic representation of the effectors was conducted using the Illustrator for Biological Sequences (IBS) tool (Xie et al., 2022).

For the prediction of the potential subcellular localization of the tomato protein candidates, the Bio-Analytic Resource for Plant Biology online tool was used (https://bar.utoronto.ca/eplant_tomato/).

For the homology search of the Sl296 and SlGLY interactors, tomato protein sequences were subjected to a broad BlastP search against non-redundant protein databases in NCBI. Sl296 homologous proteins displaying >57% identity (e-value 2e – 95) and SlGLY >55% identity (e-value 2e – 47) were retained and a multiple alignment was conducted using the NCBI MSA Viewer 1.25 online tool (**Supplementary Figure 3**). Amino acid residues are highlighted in agreement with the BLOSUM80 matrix.

### 2.2 Plant germination, growth, and AM inoculation

*Solanum lycopersium* cv MoneyMaker seeds were surface sterilized by soaking in 2.35% (w/v) sodium hypochlorite for 5 min and rinsing three times with sterile water. *Medicago truncatula* Jemalong A17 seeds were sterilized as described (Mortier et al., 2010). Both plant seedlings were grown vertically on Petri dishes with full Murashige and Skoog (MS) agar medium supplemented with vitamins for 28 days at 24°C under long-day conditions (16-h light/8-h dark photoperiod, 60% humidity).

Positively transformed *S. lycopersicum* and *M. truncatula* composite plants were transferred to 1.5-l round pots containing sterilized sand:vermiculite mixture (1:1 v/v) and plants were inoculated with approximately 250 spores of *R. irregularis* DAOM197198 (SYMPLANTA GmbH & Co. KG, Darmstadt, Germany) and supplied twice a week with 50mL of Hewitt solution (Hewitt, 1966) containing 25% of the standard phosphorus concentration. Plants were grown at 22°C under long-day conditions (16-h light/8-h dark photoperiod) and 60% of relative humidity.

### 2.3 Generation of constructs

All constructs for overexpression of effector fusion proteins were produced via Golden Gate technology. For N-terminal effector fusion constructs, the designated *R. irregularis* effector CDSs without the predicted SP (CDSΔSP) were PCR amplified from mycorrhized tomato cDNA using Q5 high-fidelity DNA polymerase and Gibson ligated into the PGGC level 0 entry vector (Addgene ID 48858) (Lampropoulos et al., 2013). For C-terminal tagged constructs, the CDSΔSP without STOP codon (CDSΔSPΔSTOP) were PCR amplified and assembled into the PGGB entry vector following the same principle (Addgene ID 48857). Green Gate level 1 modules were generated via Green Gate cloning (Lampropoulos et al., 2013) or Gibson assembly (Gibson et al., 2009) and confirmed by Sanger sequencing. Verified level 1 modules containing the effector CDS in the PGGB or PGGC were combined with level 1 modules carrying the SP, GFP CDS, linker, t35S and the PGGF screening module into the destination vector PGGPAG through Golden Gate technology (Decaestecker et al., 2019).

To monitor *M. truncatula* plant transformation, a fluorescent screening module was built by assembling Green Gate level 1 modules containing the RolD promoter, the mRuby CDS, an NLS sequence, the 35S terminator, and the F-linker-G into the destination vector PGGPAG (Decaestecker et al., 2019). The resulting construct *RolDp:mRuby : NLS:t35S* was PCR amplified with specific primers, gel purified, and Gibson assembled into a Green Gate PGGF level 0 module (Gibson et al., 2009) to generate the PGGF screening module. All primer sequences for cloning are listed in **Supplementary Table 2**.

### 2.4 Stable Arabidopsis transformation, growth and phenotypical analysis

Stable homozygous *Arabidopsis thaliana* (L.) Heynh., accession Columbia-0 (Col-0) lines carrying the effector fusions *GFP-GLOIN707* (*GLOIN707.6* and *GLOIN707.11*), *GFP-GLOIN781* (*GLOIN781.2* and *GLOIN781.12*), *GLOIN261-GFP* (*GLOIN261.5* and *GLOIN261.13*), and *GFP-RiSP749* (*RiSP749.1* and *RiSP749.2*) under control of the 35S promoter (35Sp) were generated through the floral dip transformation method (Clough and Bent, 1998). Transgenic seeds were selected based on the fluorescence‐accumulating seed technology system as described (Shimada et al., 2010). Single-locus insertions were selected at the T2 population and experiments were performed with the homozygous T3 generation.

Arabidopsis seeds were surface sterilized using chlorine gas and stratified for 48 h at 4°C in dark conditions. For the phenotypic analysis, seeds were grown vertically on agar plates containing ½ MS medium. Lateral root density (LRD) and primary root length (RL) were analyzed from the root systems of 11 or 14 days old plantlets grown vertically at 21°C under long day conditions (16/8-hours photoperiod) and 60% humidity. Root systems were photographed, and pictures were analyzed with the NeuronJ plugin using ImageJ software to determine the RL (Trujillo-Hernandez et al., 2020). LRD was calculated by dividing the number of lateral roots by the RL as described (Villaécija-Aguilar et al., 2021). Rosette pictures of 21 days old plants grown horizontally as described earlier were analyzed in image J to obtain the projected rosette area.

### 2.5 Generation of *M. truncatula* and *S. lycopersicum* composite plants and growth

Sectioned seedlings were infected by coating the freshly cut surface with an agar culture of *A. rhizogenes* Arqua1 carrying the vectors 35Sp : *GFP-GLOIN707*, 35Sp : *GFP-GLOIN781*, 35Sp : *GLOIN261-GFP*, 35Sp : *GFP*, 35Sp : *RiSP749-GFPTurbo* and 35Sp : *GFPTurbo* for *M. truncatula* and *A. rhizogenes* K599 carrying *SlPT4*p:*GFP* for *S. lycopersicum*. Transformed seedlings were grown vertically on square Petri dishes with MS agar medium supplemented with vitamins for 4-5 weeks at 24°C under long-day conditions. Plantlets were screened weekly for constitutive red fluorescent protein signal (mRuby : NLS/RFP) under the fluorescence microscope and wild-type (WT) roots were removed. The mRuby : NLS screening module allows an easy selection of transformed composite roots by red fluorescence as it is included in the same T-DNA as our GFP fusions of interest. Positively transformed composite plants were transferred to pots after a minimum of 4 weeks (Mortier et al., 2010; Ho-Plágaro et al., 2018). Composite plants were grown and inoculated as mentioned earlier. Samples for mycorrhization were collected at 4 weeks post inoculation for further analysis.

### 2.6 RNA isolation and RT-qPCR analysis

Total mRNA was extracted from ground root tissue with the ReliaPrep™ RNA Miniprep Systems (Promega) following the manufacturer’s instructions. For single-stranded cDNA synthesis, the iScript cDNA Synthesis Kit (Bio-Rad) was used. Real-time quantitative reverse transcription PCR (RT-qPCR) reactions were done with the Lightcycler 480 system (Roche Diagnostics) and analyzed with the Fast SYBR Green Master Mix (Applied Biosystems) at a final concentration of 0.25 µM for each primer. *MtGAPDH* (MTR_3g085850), *SlGAPDH* (Solyc05g014470.2), *SlEF1* (Solyc06g009960), *AT2G37620*, *AT1G13320*, *AT5G62690* were used as reference genes for *M. truncatula*, tomato, and Arabidopsis, respectively. Expression of the reference genes were stable across different experimental conditions, replicates, and tissues. Relative fold changes were calculated according to the 2^-ΔΔCt^ method (Livak and Schmittgen, 2001) after normalization against the respective reference genes and relative comparison. All RT-qPCR primers used can be found in **Supplementary Table 3**.

### 2.7 RNA sequencing of hairy root cultures

For transcriptome analysis, constructs were generated via Gateway cloning by recombining *EN-RPS5α_XVE*, open reading frames (ORFs) (effectors or GFP with attB1 and attB2 overhangs) in pDONR221, and *pEN-TurboID-flag* in the Gateway-compatible binary vector pKCTAP. All destination vectors were transformed into the *Agrobacterium rhizogenes* ATCC15834 strain and tomato hairy roots were transformed as previously described (Ron et al., 2014; Gryffroy et al., 2023). For each effector, 3-week-old root cultures of four independent transformations were treated with 100 µM β-estradiol for 24 h, harvested, crushed for RNA extractions and subjected to RNA sequencing. These cultures only consist of roots and do not have a WT shoot, which is a different system than the composite plants used for mycorrhization experiments.

To obtain gene counts from raw reads on the public usegalaxy.be servers (The Galaxy Community, 2022), we evaluated the overall quality of the reads with FastQC (default parameters), quality-trimmed the reads and clipped them with the Trimmomatic software (settings: SLIDINGWINDOW:4:20 MINLEN:80) (Bolger et al., 2014; Patro et al., 2017). The obtained high-quality reads were mapped against the tomato genome annotation ITAG2.3 and indexed by Salmon quant tool (default settings), resulting in transcript counts (http://www.bioinformatics.babraham.ac.uk/projects/fastqc/). Finally, the transcript counts were processed with tximport providing gene counts (Love et al., 2018), of which the differential expression was analyzed with the R software package (Robinson et al., 2010). The genes with an expression value higher than 0.20 counts per million (cpm; corresponding to five read counts) in at least three samples were retained for analysis. Trimmed Mean of M-values normalization was applied by the calcNormFactors function. A quasi-likelihood negative binomial regression model with effector as single factor was applied to the normalized cpm data, followed by pairwise comparisons between effectors and GFP as control. Calculated *P* values were adjusted for multiple testing (Benjamini and Hochberg, 1995).

### 2.8 Subcellular localization analysis

*Agrobacterium tumefaciens* C5851 strains carrying the vector of interest were grown in liquid YEB with the corresponding antibiotic and kept overnight at 28°C under shaking conditions. Cultures were centrifuged at 2.500 rcf for 10 min and the bacterial pellets were washed and resuspended in infiltration buffer (9.76 g/L MES, 4.76 g/L magnesium chloride, 0.98 g/L Acetosyringone; pH 5.6), mixed 1:1 to a final optical density (OD600) of 1 and injected in the leaves as described (Boruc et al., 2010)An Agrobacterium strain harboring a P19 viral suppressor of gene silencing was coinfiltrated to boost protein expression (Voinnet et al., 2015). For the plant subcellular localization assay, the effectors lacking the SP were N- and C-terminally GFP-tagged and single protein subcellular localization was investigated after infiltration. For subcellular colocalization of tomato preys with effector baits, the tomato prey CDSs were N-terminally fused to cyan fluorescence protein (CFP) via LR recombination into the pk7wgc2 destination vector and co-infiltrated with the vector containing the effector-GFP fusion proteins lacking the endogenous SP. Individual plasmid infiltration in *Nicotiana benthamiana* leaves was performed as described above for the single-protein subcellular localization of the genes. Plant material was imaged 2 days post infiltration with a 710 inverted confocal microscope (Zeiss). Primers used for the generation of these constructs can be found in **Supplementary Table 2**.

### 2.9 Estimation of *R. irregularis* root colonization

To visualize mycorrhizal structures, plant host root systems were harvested at 4 weeks post inoculation and stained as described (Demchenko et al., 2004). Colonization frequency (F%), colonization intensity (M%), root fragment colonization intensity (m%), arbuscular abundance (A%), and arbuscular abundance in mycorrhized root fragments (a%) were measured by means of the Mycocalc software (https://www2.dijon.inrae.fr/mychintec/Mycocalc-prg/download.html) as described (Trouvelot et al., 1986). For each biological repeat, a minimum of 30 inked root pieces from each line were analyzed under the light microscope.

### 2.10 Yeast two-hybrid (Y2H) library screening

The Y2H cDNA library was screened as previously described (Erffelinck et al., 2018). The pDONR221 containing the CDS of the effectors lacking their predicted SP was recombined into the PGBKT7 bait vector via the Gateway technology (Invitrogen). To exclude bait autoactivation, the PGBKT7 vectors containing the effector protein fusions were independently cotransformed with the empty PGADT7 prey vector into the reporter *Saccharomyces cerevisiae* strain PJ69-4α by the standard lithium acetate/single-stranded carrier DNA/polyethylene glycol method (Cuéllar Pérez et al., 2013). The PGBKT7 bait-competent *S. cerevisiae* was individually transformed with the in-house PGADT7 tomato root cDNA library. The cDNA library was generated from RNA extracted and pooled from 4-week-old MoneyMaker tomato plants treated with 500 µM salicylic acid for 2 h and 1 day, treated with 50 µM jasmonate for 2 h, 1 and 2 days, and treated with WT K599 OD0.1 for 2, 6 and 8 days (Ceulemans, 2021). Ten *S. cerevisiae* colonies, *i.e*., positive putative interactors, were selected from the SD/-LTH selective medium (26.7 g l^-1^ synthetic-defined medium, 0.62 g l^-1^ drop-out mix without leucine, tryptophan, and histidine [Clontech], 2% [w/v] agar) containing 5 mM of 3-amino-1,2,4-triazole (3-AT) for each bait. Their plasmid DNA was extracted, Sanger sequenced, and the prey DNA sequences were blasted against the SolGenomics and NCBI tomato genomic databases. For detection of background interactions, *S. cerevisiae* carrying the empty PGBKT7 were screened against the tomato cDNA library. The potential effector-plant protein binary interaction was assessed by Y2H pairwise assays. Plant CDSs were PCR amplified with Q5 High-Fidelity DNA Polymerase (New England Biolabs), cloned into pDONR207, and recombined into the prey vector PGADT7. Bait and prey were cotransformed in the reporter *S. cerevisiae* strain PJ69-4α as described (Cuéllar Pérez et al., 2013). Serial dilutions of transformed *S. cerevisiae* were dropped on control SD/-LT control medium, on SD/-LTH and on SD/-LTH supplemented with 5mM 3AT for 3 days at 30°C.

### 2.11 Yeast secretion trap (YST)

The cDNA sequences coding for the putative SP, the effector CDS lacking the SP, and the full-length (FL) effector were PCR amplified using adaptor primers containing the *Eco*RI and *Not*I restriction sites. The subsequent PCR products and the pYST1 destination vector containing the *SUCROSE2* (*SUC2*) invertase gene lacking its endogenous SP were further digested, ligated, and transformed into the *SUC2*-deficient *S. cerevisiae* Y02321 reporter strain (Euroscarf, Oberursel, Germany) as described (Lee et al., 2006). As a negative control for the YST assay, *S. cerevisiae* Y02321 transformed with the empty pYST1 destination vector were used, while those transformants carrying the CDS of the *M. truncatula* CLAVATA3/ESR (CLE)-related protein 13 (*MtCLE13*) fused to the *SUC2* were employed as positive control. *MtCLE13* is a peptide which is systemically transported to the root to monitor autoregulation of nodulation (Mortier et al., 2010). CLE13 has a signal peptide for secretion, a feature that is typical for CLE peptides (Murphy et al., 2012). Primers used for the generation of these constructs can be found in **Supplementary Table 2**. Transformed *S. cerevisiae* colonies were selected on SD/L, followed by a DNA insertion verification by PCR amplification, and serially diluted into control SD/L and sucrose-selective YNB/LS (6.7 g l^−1^ yeast N base without AAs, 0.69 g l^−1^ drop-out without leucine [Clontech], 2% [w/v] sucrose, and 2% [w/v] agar) agar media. Protein secretion was assessed after incubation of colonies at 30°C for 3 days.

### 2.12 Ratiometric bimolecular fluorescence complementation (rBiFC) assay

To verify the *in planta* interaction between bait and preys, we cloned the respective CDSs without stop codon into pDONR221 and in the 2in1 N-terminal rBiFC expression clones by combining the tomato preys with their respective effector proteins as described (Grefen and Blatt, 2012). All studied genes were fused in the same N-terminal positions to avoid tag interference. As negative controls, we used GLOIN707 and GLOIN781 combined with SlUNK which is considered as a background protein obtained from several in-house available Y2H screenings on the tomato cDNA library, while for RiSP749 we used LOC959 as this interaction was weak in the Y2H but could not be confirmed with rBiFC. Expression of both SlUNK and LOC959 in *N. benthamiana* leaf epidermal cells was a prior validated. An LSM 710 confocal microscope (Zeiss) with white-light laser and a 40×/1.2 water-immersion objective was used to obtain fluorescent images. Images were acquired in sequential mode, with excitation at 513 nm and 555 nm and an emission window between 519 and 550 nm and 578 and 620 nm for yellow fluorescent protein (YFP) and for RFP detection, respectively. All images were taken with the same settings. After the plant cell nucleus had been delimited by the round contour tool of the ZEN 3.5 blue edition software, the average fluorescent intensities of the RFP and YFP channels were selected. All images were devoid of saturated pixels (Yperman et al., 2021).

### 2.13 Knock-down of tomato preys with RNA interference (RNAi)

For the RNAi assay, 150-base pair fragments were PCR amplified from tomato root cDNA. Primers used for the generation of these constructs can be found in **Supplementary Table 2**. The DNA region between nucleotides 61 and 211 and nucleotides 42 and 192 was cloned and PCR amplified for the *Sl296* and *SlGLY* RNAi fragments, respectively. Purified DNA fragments were ligated into pDONR207 and subsequently recombined into the pK7GWIWG2(II)-RedRoot destination vector (Karimi et al., 2002) via Gateway technology to generate the 35Sp : *Sl296* RNAi and the 35Sp : *SlGLY* RNAi hairpin constructs. As a negative control, composite plants carrying the empty pK7GWIWG2(II)-RedRoot vector were used.

### 2.14 Protein extraction and western blot analysis

Proteins were extracted from plant material with extraction buffer (150 mM Tris-HCl, pH 7.5, 150 mM NaCl, 10% [v/v] glycerol, 10 mM EDTA, 1 mM sodium molybdate, 1 mM NaF, 10 mM dithiothreitol, 1% [v/v]) NP-40, 0.5% [v/v] polyvinylpolypyrrolidone, protease inhibitor). Total protein content was determined with the Qubit protein assay kit (Invitrogen). Total proteins were separated on 4-12% gradient Mini-PROTEAN stain-free TGX gels (Bio-Rad) and transferred to a polyvinylidene difluoride membrane. Isolated proteins were detected by immunoblot analysis with monoclonal rabbit GFP-HRP antibody (Abcam) at a 1:2000 dilution and the signal was identified through chemiluminescent substrates from the Western Lightning^®^ Plus Enhanced Chemiluminescence kit (PerkinElmer) under a ChemiDoc Imaging system (Bio-Rad).

## 3 Results

### 3.1 *In silico* selection and validation of four putative nuclear-localized effectors

To find putative plant nuclear-localized effectors secreted by *R. irregularis*, we mapped the 338 effectors identified by Zeng et al. (2018) with the effectome database of the *R. irregularis* DAOM 197198 genome (taxid:747089) (Sędzielewska Toro and Brachmann, 2016; Kamel et al., 2017) and searched for genes encoding effectors predicted to carry an NLS and an N-terminal SP and potentially excluded from the apoplast, resulting in a large list from which we only selected those that are expressed in mycorrhized roots and for which we could retrieve cDNA clones, resulting in four putative effectors, *i.e.*, GLOIN707, GLOIN781, GLOIN261, and RiSP749 (see Materials and Methods; **Supplementary Figure 1**). Full effector protein sequences were blasted against the latest *R. irregularis* proteome annotation RIR17 to rule out any mis-annotation (Maeda et al., 2018). While no AA mismatches were identified between the annotated and the investigated protein sequences of the effectors GLOIN707 and GLOIN261, the putative region PKH (position 68 to 70) located within the predicted NLS of the effector GLOIN781 was missing. For RiSP749, SignalP v4.1, the tool version we used at the start of this project, predicted an SP (likelihood 0.522), whereas SignalP v5.0 did not.

**Figure 1:**
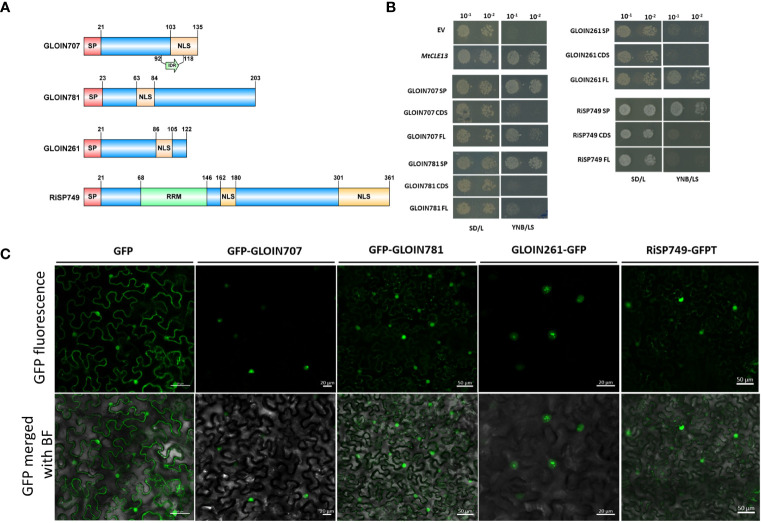
Secretion of nuclear-localized effectors GLOIN707, GLOIN781, GLOIN261, and RiSP749. **(A)** Schematic representation of the different effector protein domains in scale. Predicted signal peptide (SP), nuclear localization signal (NLS), RNA recognition motif (RRM), and intrinsically disordered region (IDR) are indicated. **(B)** Yeast Secretion Trap (YST) experiment conducted with the three different effector sequence parts, *i.e*., SP, the coding sequence without SP (CDS), and the full-length with SP (FL) fused to the *SUC2* gene in the pYST1 vector. As a negative and positive control for secretion, the empty vector (EV) and MtCLE13 were used, respectively. Transformed Y02321 *S. cerevisiae* were diluted and grown on SD/L control growth medium and on YNB/LS sucrose-selective medium for 3 days at 30°C. **(C)** Confocal images of *N. benthamiana* cells transiently expressing GFP and GFP-tagged effector fusion proteins 48 h post infiltration. Top, GFP field; bottom: overlay of GFP and bright field. At least three independent biological repeats were performed demonstrating consistent localization.

*GLOIN707* encodes a 135-AA protein containing an intrinsically disordered region (IDR) partially overlapping with the putative bipartite C-terminal NLS (**Figure 1A**; **Supplementary Figure 1**). *GLOIN781* and *GLOIN261* encode effectors of 203 AAs and 122 AAs, respectively, with only one NLS motif and no specific domains (**Figure 1A**; **Supplementary Figure 1**). Finally, *RiSP749* encodes an RNA recognition motif (RRM)-containing effector protein larger than the other candidates and carrying two NLS motifs located toward the middle and at the C-terminal region of the effector protein (**Figure 1A**; **Supplementary Figure 1**). Protein homology searches using the investigated effector protein sequences followed by further selection for secreted nuclear effector-like homologs revealed homology for GLOIN707, GLOIN781, and GLOIN261 only with putative effectors from AMF species with uncharacterized domains (**Supplementary Figure 2**). Homologous proteins of RiSP749 were also predicted to be nuclear effectors, although not all containing SPs, and were highly similar to the small 35-kDa nuclear ribonucleoprotein protein of *R. irregularis* (Rhiir259g00100) U11/U12 and to hypothetical RNA-binding domain-containing proteins without SP of various AMF (i.e *Diversispora epigaea*) and other fungi (i.e the filamentous fungus *Basidiobolus meristosporus*) (**Supplementary Figure 2**).

The potential secretion of the effectors was tested in *S. cerevisiae* by means of the YST assay (Lee et al., 2006). cDNA regions coding for the predicted SP, CDS, and FL were N-terminally fused to the *SUC2* invertase and transformed into an *S. cerevisiae SUC2*-deficient strain Y02321, which is unable to metabolize sucrose in the absence of a functionally secreted SUC2 invertase. In case the SP peptide is active, yeast growth is expected within those cells transformed with the SP and FL but not with the CDS without SP. This growth pattern was indeed obtained for positive control *M. truncatula* protein CLAVATA3/ESR (CLE)-related protein 13 (MtCLE13; Mortier et al., 2010), as well as forGLOIN707 and GLOIN781 (**Figure 1B**). Although growth of transformed cells overexpressing the *RiSP749-SUC2* SP was observed, growth of cells carrying the *RiSP749-SUC2* CDS (without SP) and FL (CDS with SP) fusion protein seemed impeded (**Figure 1B**). Also, no growth on selective medium was detected for cells carrying the CDS (without SP) and the SP alone of the effector GLOIN261, while the FL protein allowed secretion (**Figure 1B**).

To confirm the *in silico* predicted nuclear localization, we transiently expressed the N- and C-terminally GFP-tagged effector candidates lacking their endogenous SP in leaves of *N. benthamiana* under the control of the 35S promoter (**Figure 1C**; **Supplementary Figure 4**). We were unable to generate an N-terminally fused protein of GLOIN261. For both N- and C-terminal constructs, the GFP signal was visible inside the nucleus of *N. benthamiana* leaf cells transiently overexpressing the four effector fusion proteins. Green fluorescence was also detected outside the nucleus of *N. benthamiana* epidermal cells for GFP N-/C-terminal tagged GLOIN781 fusions (**Figure 1C**; **Supplementary Figure 4**).

Taken together, GLOIN707, GLOIN781, GLOIN261, and RiSP749 can potentially be secreted outside the fungus and be localized in the plant nucleus.

### 3.2 *GLOIN707, GLOIN781, GLOIN261*, and *RiSP749* are expressed during mycorrhization in tomato

**Figure 2:**
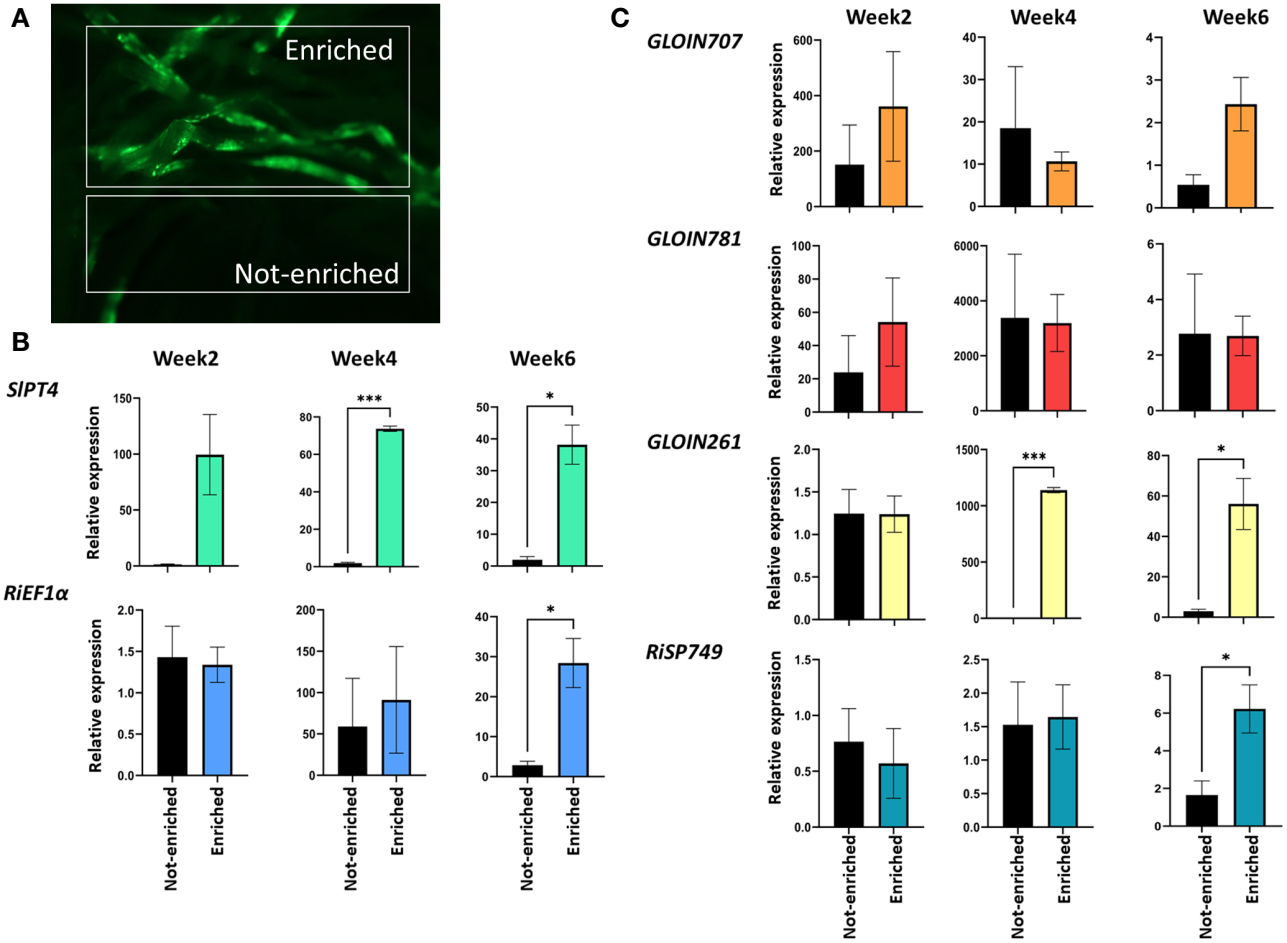
*GLOIN707, GLOIN781, GLOIN261*, and *RiSP749* expression in arbuscule-containing root regions. **(A)** Representative images of root systems of tomato composite plants expressing the *SlPT4p:GFP* construct subjected to mycorrhization. Samples taken for expression analysis are arbuscule-containing root sections (enriched) and arbuscule-depleted regions (not-enriched). **(B, C)** Expression analysis of the **(B)** fungal *RiEF1α* and the plant *SlPT4* marker genes, and **(C)** effectors in enriched and not-enriched roots. Samples were normalized with *SlEF1* and relatively compared to not-enriched regions. Data are means ± SEM of three independent biological repeats (*P < 0.05, ***P < 0.001; Student’s *t*-test).

To decipher the role of the four nuclear effectors in AM symbiosis, we first studied their gene expression pattern during mycorrhization in tomato. Because mycorrhization is restricted to specific parts of the root system, we used *SlPT4*p*:GFP*, a well-known phosphate transporter that is a marker for functional AM symbiosis, to enrich root material for arbuscule-containing cells (Harrison et al., 2002). Roots from 4-week-old tomato composite plants expressing the *SlPT4*p:*GFP* construct were inoculated with *R. irregularis* and subjected to mycorrhization for 2, 4 and 6 weeks, and arbuscule-containing (enriched for *GFP*-expressing root regions) and arbuscule-depleted (not-enriched, other non-fluorescent parts) root sections were collected under the fluorescence microscope (**Figure 2A**). *SlPT4* transcript levels were significantly increased in enriched compared with not-enriched root tissues at 4 and 6 weeks (**Figure 2B**), demonstrating that the enrichment procedure was successful. *RiEF1α*, a marker for AMF abundance, was only significantly increased for enriched samples at 6 weeks post inoculation (**Figure 2B**), demonstrating a high fungal colonization at that timepoint. Effector expression was compared relatively to the tomato *SlEF1* housekeeping gene (**Figure 2C**) or the fungal *RiEF1α* gene (**Supplementary Table 4**). Using *SlEF1* as reference, the expression levels of *GLOIN707* and *GLOIN781* were not significantly different in enriched compared with not-enriched root tissues at 2, 4 and 6 weeks (**Figure 2C**), indicating a general expression during mycorrhization in enriched and not-enriched root tissues. At 4 and 6 weeks, *GLOIN261* transcript levels were significantly higher in enriched root tissues than in not-enriched root tissues. Also *RiSP749* expression significantly increased in enriched root tissues at 6 weeks compared with not-enriched root tissues (**Figure 2C**), demonstrating a high expression of *GLOIN261* and *RiSP749* in arbuscule-containing regions. With *RiEF1α* as normalization reference, we demonstrate that all effectors are expressed in enriched and not-enriched mycorrhized root tissues (**Supplementary Table 4**).

We can conclude that the four putative effector proteins are expressed during mycorrhization in tomato, suggesting a possible function during the process.

### 3.3 Impact of *in planta* overexpression of the effector genes in symbiotic and non-symbiotic hosts

**Figure 3:**
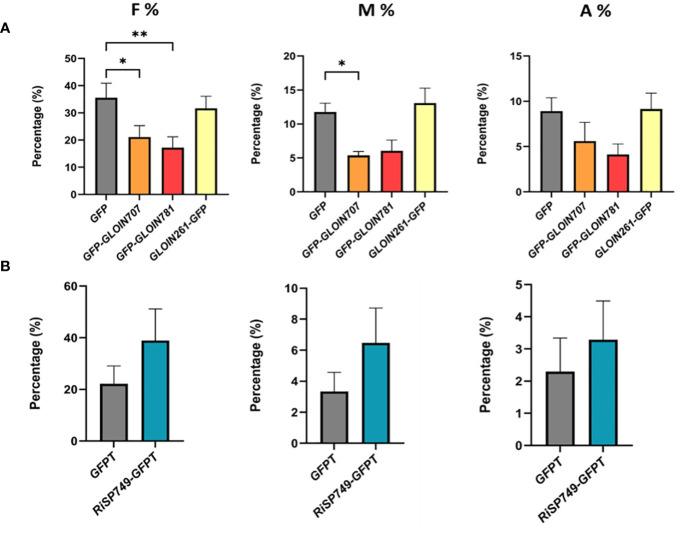
Impact on AM symbiosis of *GLOIN707, GLOIN781, GLOIN261* and *RiSP749* ectopic expression in *M. truncatula* roots. **(A, B)** Mycorrhization levels determined in *M. truncatula* transgenic roots expressing *GFP* (control), *GFP-GLOIN707*, *GFP-GLOIN781* and *GLOIN261-GFP* **(A)** and *GFPTurbo* (*GFPT*, control) and *RiSP749-GFPTurbo* (*RiSP749-GFPT*) **(B)** according to the method of Trouvelot et al. (1986). Quantification parameters refer to frequency of mycorrhiza in the root system (F%), intensity of the mycorrhizal colonization in the root (M%), and arbuscule abundance in the root system (A%). Values are means ± SEM of six **(A)** [*P < 0.05; **P < 0.005; one-way ANOVA accompanied by multiple comparison (α < 0.05)] or three to eight **(B)** biological repeats (Student’s t-test, P > 0.05).

Fungal effectors that act inside plant cells are expected to alter host physiology or immunity to allow fungal colonization. We hypothesized that ectopic expression of effector genes *in planta* might modulate the symbiosis. We therefore generated tomato composite plants with transgenic roots carrying the GFP-tagged effectors (CDS without SP) driven by the 35S promoter. Despite several attempts, we were unable to obtain tomato composite plants with effector-overexpressing roots, possibly due to silencing of the fungal gene, and therefore switched to *M. truncatula*, which is also a host for *R. irregularis.* Effector-GFP fusion transcript levels were confirmed in the respective transgenic roots (**Supplementary Figure 5A**); protein expression was clearly confirmed for *GFP-GLOIN707* and *GFP-GLOIN781* while protein levels were low for *GFP-GLOIN261* and *RiSP749-GFPT* (**Supplementary Figure 5B**). For fungal structure detection, *M. truncatula* transgenic roots were ink stained (**Supplementary Figure 6A**) and scored for mycorrhization parameters 4 weeks post inoculation (**Figure 3**). The colonization frequency (F%) of the plants overexpressing *GFP-GLOIN707* and *GFP-GLOIN781* was significantly lower than that of mycorrhized control plants and the colonization intensity (M%) was also decreased in roots overexpressing *GFP-GLOIN707* (**Figure 3A**), whereas none of these two mycorrhization parameters were affected for ectopic overexpression of *GLOIN261-GFP* and *RiSP749-GFPT* (**Figures 3A, B**). The arbuscular abundance (A%) was also not affected for the four effectors (**Figure 3**).

We next tested whether *RiEF1α* and *MtPT4* expression, markers for fungal colonization and functional symbiosis, respectively, were affected in *GLOIN707-GFP*- and *GLOIN781-GFP*-overexpressing roots 4 weeks post inoculation, but this was not the case (**Supplementary Figure 6B**). Thus, ectopically overexpressing putative fungal effectors in composite plants had no or a slight impact on the colonization and symbiosis. Surprisingly, the small impact caused by *GLOIN707* and *GLOIN781* expression was negative, which is unexpected given the positive role we expect for these effectors during symbiosis.

**Figure 4:**
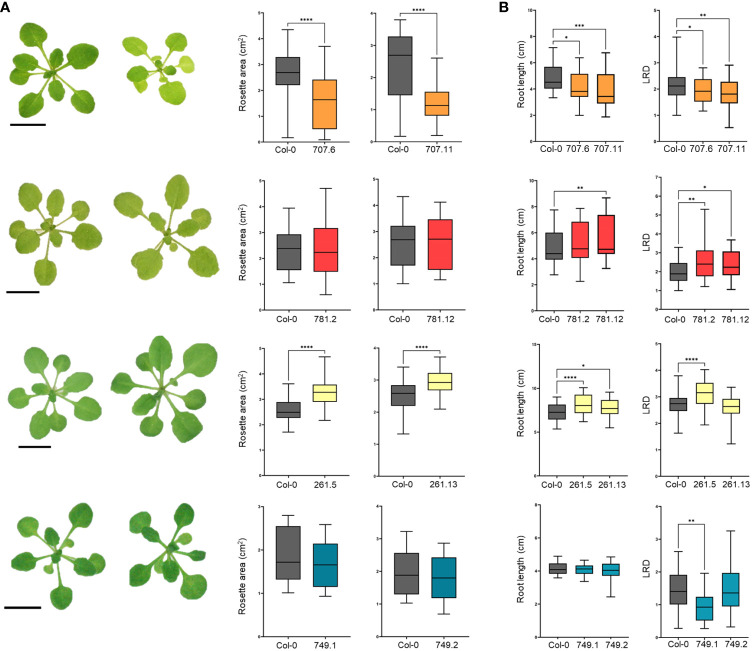
Differential modulation of Arabidopsis growth by GLOIN707, GLOIN781, GLOIN261, and RiSP749. **(A)** Representative images of rosettes and projected rosette areas of Arabidopsis WT (Col-0) control plants (left rosette) and transgenic lines (right rosette) measured at 21 DAS. **(B)** Primary root length (cm) and lateral root density (LRD, number of roots/root length) measurements of Arabidopsis transgenic lines and the WT measured at 14 DAS for GLOIN707, GLOIN781, and GLOIN261, and at 11 DAS for RiSP749. Values are means of three to four biological repeats from two independent stable Arabidopsis lines expressing the effector fusions [*n* = 12-30; *P < 0.05; **P < 0.005; ***P < 0.001, ****P < 0.0001; one-way ANOVA followed by multiple comparison (α < 0.05)].

Generating composite plants with transgenic roots allows a rapid determination of the effects on arbuscular mycorrhization but not a correct phenotyping of plant growth and development because of the heterologous expression of T-DNA genes inside the root systems only and the excessive plant handling during the procedure. Hence, stable transgenic plants should be analyzed but this is not straightforward for tomato and *M. truncatula*. Hypothesizing that effectors might target conserved plant developmental pathways, we therefore decided to express the effectors in the model plant Arabidopsis, which is not an AMF host but easily transformable. Two independent homozygous stable Arabidopsis lines ectopically expressing each effector were generated and subjected to phenotypical analysis. RT-qPCR confirmed high transcript levels and GFP fluorescence images validate their expression in Arabidopsis roots (**Supplementary Figure 7**). The consequence of effector overexpression on Arabidopsis shoot growth was defined by measuring the projected rosette area at 21 days after sowing (DAS). The rosette area of the *GFP-GLOIN707* and *GLOIN261*-*GFP* lines was significantly smaller and larger, respectively, than that of the WT, but remained unchanged for the *GFP-GLOIN781* and *GFP-RiSP749* lines (**Figure 4A**). Compared with the WT, the primary root length (RL) and lateral root density (LRD) were significantly reduced in both *GFP-GLOIN707* lines, but LRD and RL were significantly increased for both *GFP-GLOIN781* and *GLOIN261-GFP* lines, respectively, whereas root growth was not consistently different in the *GFP-RiSP749* lines (**Figure 4B**). Thus, ectopic expression of *GLOIN707* prevents normal shoot and root growth in Arabidopsis, in contrast to *GLOIN261*, which seems to promote shoot and root growth, whereas *GLOIN781* seems to affect LR formation and *RiSP749* has no impact on plant development.

Together, ectopic expression of *GLOIN707* seems to have a negative impact on plant development and slightly decreases the symbiosis outcome. On the other hand, ectopic expression of *GLOIN261* and *GLOIN781* caused an increase in root growth parameters in Arabidopsis while ectopic expression of *GLOIN781* but not *GLOIN261* had a slight negative impact on the symbiosis. The ectopic expression of *RiSP749* had no effect on the symbiosis, nor on Arabidopsis plant development.

### 3.4 Searching effector-interacting host tomato proteins

**Figure 5:**
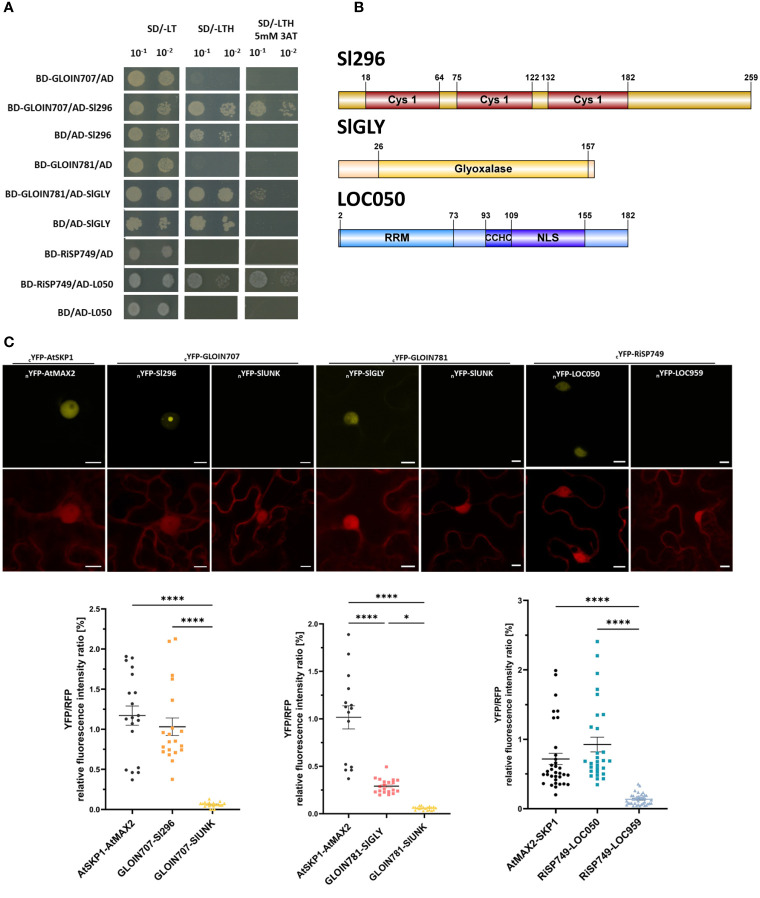
Protein–protein interactions between the effectors and tomato plant proteins. **(A)** Binary Y2H assays confirming the interaction of GLOIN707 (BD-GLOIN707) with the tomato protein Sl296 (AD-Sl296), GLOIN781 (BD-GLOIN781) with SlGLY (AD-SlGLY), and RiSP749 (BD-RiSP749) with LOC050 (AD-L050) in selective medium supplemented with 5 mM 3-AT. As negative control, the tomato preys were cotransformed with the empty PGBKT7 vector (BD/+) and the effector protein baits with the empty PGADT (+/AD). **(B)** Schematic representation of the different protein domains of the tomato preys Sl296, SlGLY and LOC050. Cys1, Cysteine/Histidine-rich C1 domain; Gly, Glyoxalase domain; NLS, Nuclear localization signal; RRM, RNA recognition motif; CCHC, CCHC-type zinc finger domain are indicated. **(C)** rBiFC assays corroborating nuclear YFP reconstitution due to protein–protein interaction between GLOIN707 and Sl296, between GLOIN781 and SlGLY, and between RiSP749 and LOC050 N-terminally split YFP fusions (top). Nuclear YFP signal was also detected in the positive control AtSKP1-AtMAX2, whereas lack of YFP signal was observed for the negative controls (nYFP-SlUNK and nYFP-LOC959). RFP fluorescent signal corresponds to the constitutively expressed control cassette (bottom). Three independent experiments were conducted and a total of 15-20 cells were analyzed. Bars = 10 µM. **(D)** YFP/RFP relative fluorescent intensity ratios of the rBIFC protein pairs from **(C)**. Data are means ± SEM of three biological replicates [*n* = 15-20; *P < 0.05; ****P < 0.0001; two-way ANOVA analysis followed by multiple comparison (α < 0.05)].

To identify possible interacting tomato protein partners of the four effectors, we performed Y2H assays with GLOIN707, GLOIN781, GLOIN261, and RiSP749 as baits (PGBKT7, -BD) and a tomato root cDNA library as prey (PGADT7, -AD). After excluding bait autoactivation, we analyzed ten single colonies grown on SD/-LTH + 5 mM 3-AT for each effector. This screening resulted in two plant protein candidates for GLOIN707, six for GLOIN781, and two for RiSP749 (**Table 1**), but none when GLOIN261 was used as bait. We validated the interactions of GLOIN707, GLOIN781, and RiSP749 with their potential tomato targets based on their predicted nuclear localization by pairwise Y2H assays (**Figure 5A**; **Supplementary Figure 8**; **Table 1**). Only the cotransformed *S. cerevisiae* containing the vector pairs BD-GLOIN707/AD-Sl296 and BD-RiSP749/AD-LOC050 interacted strongly on selective medium SD/-LTH + 5 mM 3-AT, and BD-GLOIN781/AD-SlGLY only weakly (**Figure 5A**). BD-RiSP749/AD-LOC959 interacted weakly, while the interaction between GLOIN707 and Sl237 is probably due to the strong auto-activity of Sl237, hence it was not taken along in the following experiments (**Supplementary Figure 8**). Protein domain analysis of Sl296, a Cys-His-Pro (CHP)-rich zinc finger protein-like protein highly homologous with many uncharacterized and hypothetical proteins of different plant species, revealed the presence of three C1 domains equally distributed along the full length (**Figure 5B**). These C1 domains are characterized by a rich cysteine and histidine content found in protein kinase C, probably involved in binding different ligands, such as diacylglycerol (Azzi et al., 1992). A broad homology search identified Sl296 proteins to be mainly present in the Solanaceae family of plants (**Supplementary Figure 3**). LOC050 encodes a serine/arginine-rich splicing factor RSZ22-like protein, homologous to many RSZ22-like proteins from various plant species. Like RiSP749, LOC050 carries an NLS and an RRM domain, involved in RNA binding (Birney et al., 1993), but also a CCHC-type zinc finger domain that may be involved in RNA, DNA or protein binding (**Figure 5B**). As SlGLY contains an N-terminal glyoxalase-VOC1 domain (**Figure 5B**), typically found in metalloenzymes, such as glyoxalase I (Armstrong, 2000), it may encode a glyoxalase I protein that detoxifies methylglyoxal (MG), a byproduct of sugar metabolic pathways, which might also be involved in signaling (Hoque et al., 2016). A protein homology search of SlGLY demonstrates that it is conserved across various plant species (**Supplementary Figure 3**).

**Table 1:** Potential tomato protein interactors of GLOIN707, GLOIN781, and RiSP749 baits identified with Y2H screen.

BAIT	PREY	DESCRIPTION	SUBCELLULAR LOCALIZATION	NICKNAME
**GLOIN707**	Solyc01g073830	CHP-rich zinc finger protein-like 237	NUCLEUS	Sl237
	Solyc01g073820	CHP-rich zinc finger protein-like 296	NUCLEUS	Sl296
**GLOIN781**	Solyc09g091000	Major allergan Mal d 1	CYTOPLASM	/
	Solyc05g012580	Unknown protein	PLASTID	/
	Solyc12g099120	Abscisic acid induced MYB transcription factor	NUCLEUS	SlMYB
	Solyc03g083390	Nuclear movement protein nudc	NUCLEUS	SlNUC
	Solyc06g007610	Lactoylglutathione lyase/glyoxalase I family protein	NUCLEUS	SlGLY
	Solyc01g099670	40S ribosomal protein S10-like	CYTOPLASM	/
**RiSP749**	LOC101266050	Arginine/serine-rich splicing factor RSZ22	NUCLEUS	LOC050
	LOC101247959	Uncharacterized N-acetyltransferase p20-like	CYTOPLASM	LOC959

Potential subcellular localization of the putative interactors was determined using the Bio-Analytic Resource for Plant Biology online tool (https://bar.utoronto.ca/eplant_tomato/).

The putative effector-interacting plant proteins Sl296, SlGLY and LOC050 should localize to the same subcellular compartments to be able to interact with the nuclear effectors. Their subcellular localization *in planta* was investigated by visualization of N-terminal CFP-tagged fusion proteins after transient expression in *N. benthamiana* leaves alone or in combination with their respective N-terminal GFP-labeled effector (**Supplementary Figure 9**). When expressed alone, the fluorescence signals of *CFP-SlGLY* and *CFP-LOC050* were restricted to the nucleus, whereas *CFP-Sl296* mainly accumulated in the cytoplasm and weakly in the plant nucleus (**Supplementary Figure 9A**). When coexpressed with the corresponding *GFP-*fused effector partners, all co-infiltrated protein pairs were detected in the plant nucleus, confirming the nuclear colocalization of GLOIN707 and Sl296, GLOIN781 and SlGLY, and RiSP749 and LOC050 (**Supplementary Figure 9B**).

To evaluate interactions *in planta*, an rBiFC assay in *N. benthamiana* leaves (Grefen and Blatt, 2012) was performed with both prey (tomato proteins) and bait (effectors) N-terminally fused to the corresponding split YFP version (nYFP) in the same 2in1 destination vector, which also contained an RFP cassette constitutively expressed under the 35S promoter, allowing fluorescence intensity normalization and equal gene dosage in each tobacco cell in which the T-DNA has been transiently inserted. In addition, as the fluorescence proteins (YFP and RFP) are driven by the same strong promotor, differences in fluorescence intensity ratios can be interpolated between reconstituted YFP and RFP. These characteristics excludes the high false positive rate observed with traditional BiFC analysis. The known nuclear interactors AtSKP1 (At1g75950) and AtMAX2 (At2g42620) (Woo et al., 2001) were used as a positive control (cYFP-AtSKP1/nYFP-AtMAX2), while as a negative control, we used GLOIN707 and GLOIN781 combined with SlUNK which is considered as a background protein obtained from in-house Y2H screenings on the tomato cDNA library, and RiSP749 with LOC959 as we could not confirm their interaction with rBIFC. For the positive controls AtSKP1-AtMAX2 and GLOIN707-Sl296, a reassembled YFP signal was detected, more specifically in the nucleolus and in nuclear speckles, and to a lesser extent for the GLOIN781-SlGLY and RiSP749-LOC050 protein pairs in the nucleus (**Figure 5C**). Relative YFP/RFP fluorescence intensity ratios indicated significant differences between the positive and the negative controls, as well as between the negative controls and cYFP-GLOIN707/nYFP-Sl296, cYFP-GLOIN781/nYFP-SlGLY, and cYFP-RiSP749/nYFP-LOC050 (**Figure 5D**), confirming a strong *in vivo* protein association between the effectors and preys in the plant nucleus. Fluorescence ratios between the positive controls cYFP-AtSKP1/nYFP-AtMAX2 significantly differed from those of cYFP-GLOIN781/nYFP-SlGLY, suggesting a weaker or a heterogenous *in vivo* nuclear protein–protein interaction.

In summary, we identified three distinct nuclear host plant protein interactors for GLOIN707, GLOIN781, and RiSP749, but not for GLOIN261.

### 3.5 Sl296 and SlGLY might be involved in tomato mycorrhization

**Figure 6:**
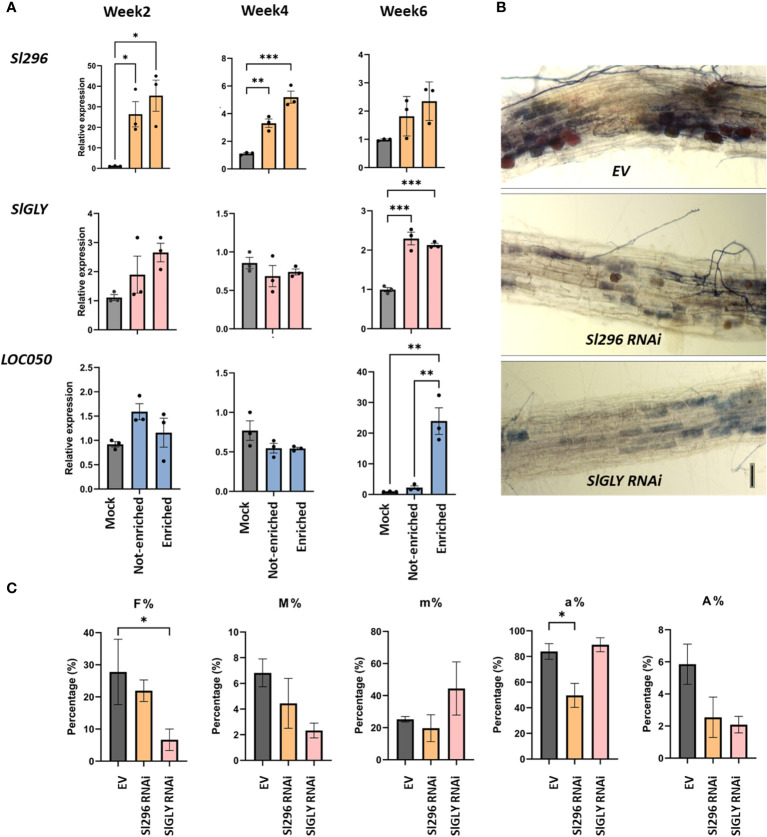
Involvement of Sl296, SlGLY and LOC050 in AM symbiosis in tomato roots. **(A)** Expression analysis of *Sl296, SlGLY* and *LOC050* in root systems of tomato composite plants expressing the *SlPT4p:GFP* construct subjected to mycorrhization for 2, 4, and 6 weeks. Samples taken for expression analysis were “Enriched” for arbuscule-containing root sections, “Not-enriched” for regions depleted in arbuscules, and “Mock” for uninoculated roots. Samples were normalized with *SlEF1* and relatively compared to Mock. Data are presented as means ± SEM of three independent biological repeats [*n* = 3-6; *P<0.05; **P < 0.005, ***P < 0.001; two-way ANOVA analysis followed by multiple comparison (α < 0.05)]. **(B)** Representative images of ink-stained EV, *Sl296* and *SlGLY* RNAi hairpin-expressing root systems. **(C)** Quantification of mycorrhization in tomato EV control roots and in *Sl296* and *SlGLY* RNAi hairpin construct lines according to the method of Trouvelot et al. (1986). Quantification parameters refer to frequency of mycorrhiza in the root system (F%), intensity of the mycorrhizal colonization in the root (M%), intensity of the mycorrhizal colonization in the root fragment (m%), arbuscule abundance in mycorrhizal parts of root fragments (a%), and arbuscule abundance in the root system (A%). Values are means ± SEM of three biological repeats [*n* = 3-6; *P < 0.05; two-way ANOVA followed by multiple comparison (α < 0.05)].

To investigate the potential role of the tomato nuclear proteins Sl296, SlGLY and LOC050 in AM, we investigated their expression profiles in specific symbiotic-enriched root segments by means of the *SlPT4p:GFP* composite plants as described for the four nuclear effectors and also included non-inoculated (mock) root segments (**Figure 6A**). Transcript levels of *Sl296* were significantly higher in *SlPT4*-enriched and not-enriched mycorrhized regions than those of mock roots at 2 weeks and, to a lesser extent, at 4 and 6 weeks. *SlGLY* and *LOC050* expression remained unchanged for mock and mycorrhized *SlPT4*-enriched and not-enriched root segments at 2 and 4 weeks. *SlGLY* significantly increased at 6 weeks in mycorrhized root segments. *LOC050* was very strongly induced in *SlPT4*-enriched root regions and not in not-enriched and mock roots at 6 weeks.

To gain further insight into the role of Sl296, SlGLY, and LOC050 in AM symbiosis, we tested mycorrhization at 4 weeks in roots of composite tomato plants showing a reduced target plant gene expression via RNAi. Despite several attempts, no *LOC050* knock-down roots could be obtained. Mycorrhized RNAi roots were analyzed by RT-qPCR to verify the downregulation of the transcript expression of *Sl296* and *SlGLY* (**Supplementary Figures 10A, B**) and mycorrhization parameters were assessed (**Figures 6B, C**). In both *Sl296* and *SlGLY* RNAi transgenic roots, mycorrhization was visibly lower than that in control roots (**Figure 6B**). Moreover, partial *Sl296* and *SlGLY* silencing significantly reduced the arbuscule abundance in mycorrhized areas (a%; **Figure 6C**) and colonization frequency (F%; **Figure 6C**), respectively. The remaining colonization parameters were unchanged. We next tested whether *RiEF1α* expression was affected in these RNAi lines by RT-qPCR analysis and could not confirm any significant changes in expression (**Supplementary Figure 10C**).

Taken together, *Sl296* is expressed upon mycorrhization at earlier symbiotic stages in a general way and its knock-down decreases the number of arbuscules in the roots, hinting at its involvement in arbuscule establishment and development. On the contrary, *SlGLY* is expressed at later stages during mycorrhization, also in a non-arbuscular specific way and its partial downregulation decreases the mycorrhization frequency. *LOC050* expression is particularly strong in root regions with functional arbuscules, but a role in growth and symbiosis could not be inferred due to missing RNAi lines.

### 3.6 Nuclear effectors trigger different transcriptional responses in tomato

**Figure 7:**
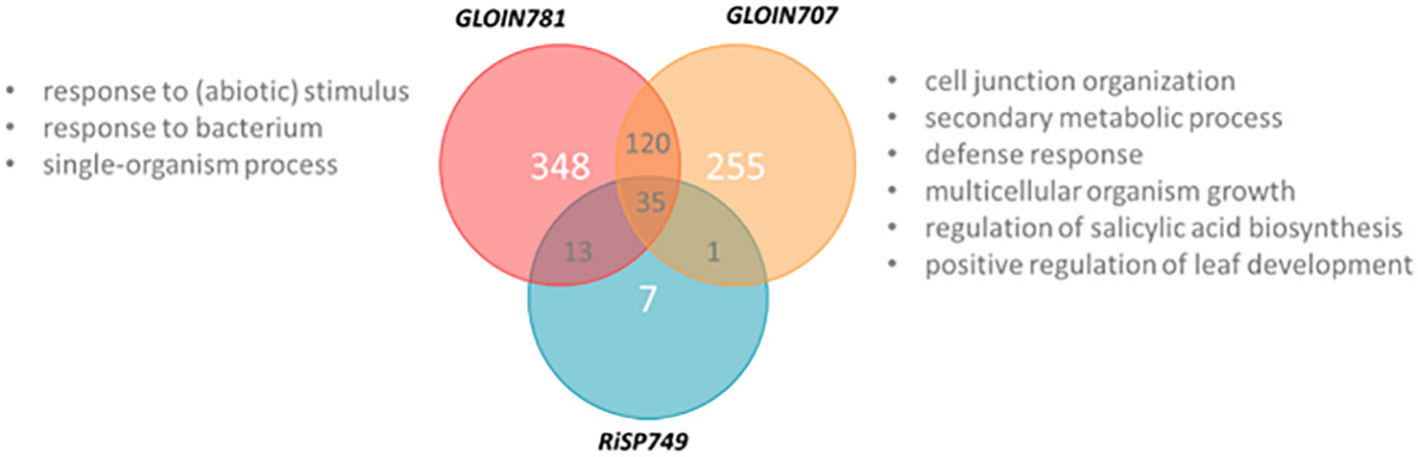
Venn diagram representing differentially expressed genes of GLOIN707, GLOIN781, and RiSP749. *XVE::GFP, XVE::GLOIN707, XVE::GLOIN781*, and *XVE::RiSP749* transgenic tomato hairy root cultures were harvested 24 h after estradiol treatment, and subjected to RNA sequencing. GO categories of differentially upregulated genes identified with PLAZA 4.5 Dicots indicated in “gray”.

As indicated above, we were not able to obtain tomato composite plants with roots constitutively expressing the effector genes when driven by the 35S promoter, possibly due to silencing of the fungal gene. Therefore, we used a construct (*RPS5α_XVE::effector CDS-TurboID-flag*) with which we could induce the effector expression via estradiol treatment of hairy root cultures starting from tomato cotyledons (Gryffroy et al., 2023). Although those root cultures are in liquid and not suitable for phenotyping experiments, they can be used to explore how the nuclear-localized effectors affect the host plant transcriptome. We analyzed the transcriptome by an RNA-sequencing experiment on tomato hairy root cultures, 24 h after estradiol treatment to induce the expression of *GLOIN707, GLOIN781* and *RiSP749* (**Supplementary Table 5**, **Supplementary Figure 11**). Compared to GFP, 411 genes were found to be differentially expressed after *GLOIN707* expression (false discovery rate [FDR]<0.05; **Figure 7**), 56 after *RiSP749* expression (FDR<0.1), and 516 after *GLOIN781* expression (FDR<0.05). Several genes were shared and after subtracting these overlaps, the resulting lists of differentially expressed genes were 255 for *GLOIN707*, 348 for *GLOIN781*, and only seven for *RiSP749* (**Figure 7**; **Supplementary Table 5**). These lists were subjected to gene ontology (GO) enrichment studies for biological processes with PLAZA 4.5 Dicots (Van Bel et al., 2018). For *GLOIN707*, upregulated genes encoding proteins related to ‘cell junction organization’ (P=1.54E-02), ‘secondary metabolic process’ (P=1.76E-02), ‘defense response’ (P=9.09E-3), ‘multicellular organism growth’ (P=1.96E-02), ‘regulation of salicylic acid biosynthesis’ (P=1.96E-02), and ‘positive regulation of leaf development’ (P=2.46E-02) were significantly overrepresented, whereas genes related to ‘mitochondria’ in the cellular component GO category were significantly depleted (P=1.62E-02). For GLOIN781, three response-related significantly enriched GO categories were found for upregulated genes, ‘response to stimulus’ (P=2.45E-03), ‘multi-organism process’ (P=2.83E-02) with the subcategory ‘response to bacterium’ (P=1.75E-02), and ‘single-organism process’ (P=3.18E-03), whereas ‘macromolecular complex’ category was depleted (P=5.23E-03). No significantly enriched categories could be found for the differentially expressed genes for RiSP749.

For each putative effector, the most highly up- and downregulated genes (approximately 15 or fewer) were subjected to RT-qPCR analyses to confirm the expression patterns against the GFP control plants (**Supplementary Figure 12**). The genes for which the pattern could be confirmed are listed in **Table 2**. For the GLOIN707-responsive genes, we validated differential expression of a diverse set of genes reflecting the diverse GO ontology categories found in the RNA-sequencing results. Besides genes involved in transport and oxidoreduction processes, two of the GLOIN781-downregulated candidate genes encoded MG-related genes, *i.e.*, glyoxylate reductase (*Solyc08g080030*) and glyoxal oxidase (*Solyc04g081130*), in line with the nature of the GLOIN781-interacting tomato protein candidate SlGLY. Six of the seven unique RiSP749-responsive genes could be confirmed with RT-qPCR, with downregulated genes coding for proteins involved in metabolic processes. In conclusion, induction of the three putative effectors in tomato roots elicited distinct transcriptional responses, suggesting that they differentially reprogram the host plant physiology.

**Table 2:** Significant differentially expressed genes 24 h after GLOIN707, GLOIN781, and RiSP749 induction confirmed with RT-qPCR analysis.

EFFECTOR	GENE ID	EXPR.	DESCRIPTION	GO
**GLOIN707**	Solyc03g026050	DOWN	self pruning 3C	flower development
	Solyc06g054060	DOWN	non-specific lipid-transfer -like protein	lipid transport
	Solyc11g020990	DOWN	proteinase inhibitor II	response to oxidative stress
	Solyc11g032060	DOWN	GDSL esterase/lipase	hydrolyse activity
	Solyc01g099880	UP	sugar will eventually be exported transporter protein	sugar transport
	Solyc04g074050	UP	Serine/threonine receptor-like protein kinase	response to stimulus or other organisms, protein kinase activity
	Solyc01g102380	UP	germin-like protein	cell-cell junction
	Solyc06g035710	UP	multidrug resistance protein mdtK	transmembrane transport
	Solyc12g009920	UP	UDP-glucuronosyl/UDP-glucosyltransferase	metabolic process
**GLOIN781**	Solyc10g050690	DOWN	copper-ion binding proteins	electron carrier activity
	Solyc10g050730	DOWN	copper-ion binding proteins	electron carrier activity
	Solyc09g005260	DOWN	calcium/proton exchanger	transmembrane transport
	Solyc02g088560	DOWN	cyclic nucleotide channel	transmembrane transport
	Solyc06g066560	DOWN	aquaporin	water transport
	Solyc00g071180	DOWN	multicystatin	cysteine-type endopeptidase inhibitor activity
	Solyc08g080030	DOWN	glyoxylate reductase	oxidation-reduction process
	Solyc04g081130	DOWN	glyoxal oxidase	galactose oxidase
	Solyc09g092600	UP	cytochrome P450 enzymes	oxidation-reduction process
	Solyc09g092590	UP	cytochrome P450 enzymes	oxidation-reduction process
	Solyc05g052600	UP	Fructose-1 6-bisphosphatase	carbohydrate biosynthetic process
	Solyc12g008900	UP	Cytokinin oxidase6	oxidation-reduction process
**RiSP749**	Solyc01g108540	DOWN	acetyl esterase	metabolic process
	Solyc12g088760	DOWN	subtilisin-like protease	serine protease
	Solyc01g111630	DOWN	glyoxylate/hydroxypyruvate reductase	oxidation-reduction process
	Solyc01g018020	DOWN	transketolase-like protein	metabolic process
	Solyc07g054770	UP	wound-induced protein	unassigned
	Solyc06g034000	UP	MYB-related transcription factor	transcription factor

Expr, expression; GO, Gene Ontology of biological processes with PLAZA 4.5 Dicots.

## 4 Discussion

Studying the involvement of fungal genes in arbuscular mycorrhization is not an easy task because the fungus remains recalcitrant to genetic modification, hampering genetic studies that are often the final proof of the protein’s functionality (Helber and Requena, 2008). AMF are expected to secrete hundreds of effectors outside or inside plant cells to change plant immunity and physiology, allowing fungal accommodation inside the plant cells to establish a functional symbiosis. Therefore, to unravel the role of possible plant nuclear-localized effectors in growth and mycorrhization, as well as their corresponding plant nuclear protein targets and transcriptional responses in tomato roots, we used different biochemical and genetic approaches, following the recently reported stepwise pipeline (Aparicio Chacón et al., 2023). These tools brought us closer to the possible function of four fungal effectors but also shed light on the associated drawbacks of the used methods.

Based on existing lists of putative effectors, we identified four candidates possibly encoding fungal effectors. We found that GLOIN707, GLOIN781 and GLOIN261 share homology with hypothetical nuclear effector-like proteins from other AMF, suggesting potential roles during AM symbiosis. RiSP749 displayed a broader homology with predicted effectors and ribonucleoproteins from AMF and other organisms. All four effectors were expressed during symbiosis in tomato, revealing a higher expression for *GLOIN261* and *RiSP749* when comparing the expression in root tissue enriched for functional arbuscules (identified through *PT4* expression) to the non-enriched root tissue. Also in the stage-specific dataset of Zeng et al., GLOIN261 demonstrated induced expression in arbuscules in *M. truncatula*, while the other effectors are not detected (Zeng et al., 2018). In the future, it would be interesting to complement these expression studies with stage-specific expression analysis specifically in tomato, by for instance state-of-the-art single-cell analysis or by *in situ* hybridization as was done for RiNLE1 (Wang et al., 2021). However, expression does not ensure that the putative effector acts intracellularly, and besides the YST assay and tagging with fluorescent proteins for subcellular localization studies that we have done in this work, future experiments such as immunolocalization (Wang et al., 2021) or (single-cell) proteomics, which is an upcoming field in effector research, should be performed (Kelly, 2020; Miltenburg et al., 2022).

Nevertheless, intracellular effectors are expected to be secreted outside the fungus and translocated inside the plant cytoplasm. All four proteins were predicted to have a functional SP, although prediction for RiSP749 differed between different versions of the SignalP tool (SignalP v4 predicted one, while SignalP v5.0 did not). Possible functionality of the SP was demonstrated using the YST system, which is an easy system based on yeast growth via an SP-driven invertase secretion (Lee and Rose, 2012). The predicted SP of GLOIN707, GLOIN781 and RiSP749 were indeed able to secrete the yeast invertase, allowing colony growth indicative for functional SPs, irrespective of mixed outcomes of the predictive programs for RiSP749. In accordance, the GLOIN707 and GLOIN781 full-length CDS also allowed yeast growth, while this was not the case for the full-length RiSP749. RiSP749 is the largest protein and hence the large size might hinder the invertase activity of the secreted fusion protein. Alternatively, the RiSP749 CDS might contain information impeding secretion. In contrast to the other three effectors, the predicted GLOIN261 SP invertase fusion was not sufficient to guarantee growth of the transformants, while the full-length version did, indicating the involvement of additional protein regions or protein structures that may be implicated in secretion, possibly via a non-conventional pathway (Stuer et al., 2023). Although the YST assay is broadly used in fungal effector research to validate effectors’ secretion, the biological context in which these effectors are secreted is far more complex and might be influenced by plant-derived molecules that are lacking in the yeast experimental set-up. Therefore, besides validating whether the proteins were properly expressed in the yeast cells, also alternative approaches such tomato roots treated with synthetic effector peptides or immunolocalization on symbiotic tissue would help to unravel the effectors internalization. Furthermore, expression in transformable tomato root-interacting fungi such as *Fusarium solani* could be tried to test their secretion and translocation *in planta* (Skiada et al., 2019).

The four effectors have been selected based on the presence of possible NLSs. Fluorescence microscopy analysis indeed confirmed the nuclear subcellular localization of all protein fusions studied, but more detailed research will be required to verify the involvement of the *in silico*-predicted NLS in the import of the effectors into the plant nucleus, because some of the fusion proteins were also detected within other subcellular compartments, such as the cytosol. It might be interesting to test whether the NLS of the investigated effectors is the sole responsible for the effector’s translocation into the nucleus via the canonical α/β-importin pathway or whether, in contrary, effectors can passively diffuse through the nuclear pores, as they display molecular weights below 40 kDa (Liu and Coaker, 2008; Harris et al., 2023).

A common analysis used to study the effect of fungal effectors on plant physiology is through ectopic expression (Kloppholz et al., 2011; Zeng et al., 2020; Wang et al., 2021), but this also has some limitations, such as the transgene may be silenced, or the observed general phenotype may not correspond to the true function due to misexpression. We generally experienced a high level of silencing when constitutively expressing the fungal effector genes in tomato, making it difficult to analyze their effect on mycorrhization in tomato composite plants. Therefore, we substituted composite plants with another host, *M. truncatula*. In addition, we used the non-host Arabidopsis, because we hypothesized that if the effector targets a conserved pathway in plant development, we might also observe the effect in this easily transformable plant, regardless of whether it is a host or not. Phenotyping the effect of the ectopic expression of the effectors and silencing of the tomato proteins on AM colonization was inferred with the Trouvelot method and via expression of *RiEF1α* and *MtPT4* expression, but no consistent conclusions could be made at the molecular level. This discrepancy might be due to the variation observed among the phenotypes of various transgenic roots with differences in their levels of effector expression, and the roots utilized for qRT-PCR analysis. Increasing the sample size, and the use of alternative approaches such as the magnified intersections method, might be helpful in the future to investigate the effect on mycorrhization in more detail (McGonicle et al., 1990).

While studying the involvement of GLOIN707 in AM symbiosis, we observed a negative effect on the growth of Arabidopsis plants constitutively expressing the *GLOIN707* effector. This reduction in plant growth may also explain the observed negative effect on the mycorrhization frequency and intensity in *M. truncatula*. This effect is rather unexpected, because effectors should contribute to symbiosis, not hinder it. Hence, these phenotypes reflect a major drawback of ectopic expression strategies and should be interpreted with caution, because observed phenotypes may be indirectly caused by general overexpression, by the fact that effectors might not act alone during symbiosis, or because they might need specific environmental conditions to exert their beneficial role in AM symbiosis. By using an inducible expression system in tomato hairy root cultures, we could investigate the effect of short-term overexpression of *GLOIN707* on the transcriptome. This analysis resulted in differential expression of more than 200 genes for GLOIN707, with a significant overrepresentation of genes involved in cell-cell junction assembly and defense response to other organisms. It is tempting to speculate that activation of defense might explain the negative effect on plant development and symbiosis, although, future experiments should validate this mode-of-action. The phenotypic and transcriptional data suggest that GLOIN707 interferes with key cellular processes in the host plant, under which response to (a)biotic factors, metabolism and transport. Another way to address the function of the effector is through reverse genetic approached to find the plant proteins with whom they interact. Y2H analysis followed by rBiFC validation revealed a strong interaction between GLOIN707 and the tomato protein Sl296, encoding a CHP zinc-finger protein-like protein that is mainly restricted to plants of the Solanaceae family, suggesting a specialized role within that family. This interaction may redirect Sl296 from the cytosol to the nucleus, where both proteins were found to associate in the nucleolus, the high activation site of ribosomal rRNA synthesis and ribosome biogenesis (Kalinina et al., 2018). Zinc-finger proteins are nuclear proteins involved in transcriptional or translation regulation of RNA, DNA or proteins upon (a)biotic stresses (Han et al., 2021). Reduced expression of *Sl296* decreased the arbuscular abundance in mycorrhized root fragments measured by microscopical imaging, suggesting that it may be required for proper arbuscular development, although we could not confirm this with *RiEF1α*. Expression of *Sl296* was AM responsive, especially at early stages, similarly to the expression of *GLOIN707*. How GLOIN707 affects the Sl296 action needs further analysis, but given the possible *Sl296* RNAi-impaired AM phenotype and the nucleolar site of the interaction, the effector may have an impact on ribosome biogenesis or rRNA transcription modulation via its interaction with Sl296 to increase the plant’s metabolism to accommodate the fungus. It would be of interest to produce stable tomato *Sl296* knock-out mutants via CRISPR technology to unequivocally confirm the observed AM phenotypes and further characterize the biological relevance of this protein association.

Although the ectopic expression of the nuclear-localized effector GLOIN781 negatively affected the mycorrhization frequency in *M. truncatula*, it had a positive effect on Arabidopsis root growth. Protein–protein interaction approaches revealed the nuclear interaction between GLOIN781 and the SlGLY protein, encoding a putative glyoxalase, present in many different plant families implying a conserved and fundamental role of GLY proteins in plant development. Indeed, glyoxalases detoxify MG, a byproduct of several metabolic pathways in plant cells that causes oxidative stress when abundant (Thornalley, 2003) and acts as a signaling molecule at low concentrations, interacting with cytosolic calcium ions (Hoque et al., 2016). Recently, two potential growth-promoting microbes, *i.e.*, *Pseudomonas* sp. CK-NBRI-02 and *Bacillus marisflavi* CK-NBRI-03, have been found to alter MG levels and, subsequently, the MG detoxification machinery in Arabidopsis to enhance plant defense responses and growth (Kaur et al., 2022). *SlGLY* expression was pronounced in mycorrhized tomato roots at late stages, *i.e*., 6 weeks, whereas *GLOIN781* was more highly expressed in mycorrhized tissues (enriched and not-enriched segments) at 2 and 4 weeks. Furthermore, partial *SlGLY* silencing appeared to negatively affect the AM colonization frequency, although we could not confirm this with *RiEF1α* expression. As two of the significantly downregulated *GLOIN781* target genes of tomato, *i.e.*, glyoxylate reductase and glyoxal oxidase, play a role in MG homeostasis, we hypothesize that GLOIN781 may regulate the nuclear MG levels through its association with SlGLY, and that this interaction might participate in the AM-dependent calcium spiking to control the initiation of symbiosis. How this regulation is achieved is currently unknown, but given that metabolic pathways are highly active during arbuscule establishment and functioning, and that both genes are expressed in colonized root fragments with or without arbuscules, a role for MG in maintaining and initiating arbuscules can be assumed and should be investigated in the future. To further confirm the potential role of GLOIN781-SlGLY interaction and MG regulation in AM, the glyoxal I enzymatic activity of SlGLY should be tested, as well as nuclear Ca2+, K+, and MG levels could be quantified in plant lines ectopically expressing GLOIN781 and in the SlGLY RNAi lines.

Our experiments did not deliver much more insight into the function of GLOIN261. The expression analysis revealed a specific enrichment in arbuscule-containing root segments at later stages, and expression of *GLOIN261* played a beneficial role in Arabidopsis root and leaf growth, although the mycorrhization parameters did not change significantly in mycorrhized composite lines of *M. truncatula*. Besides its nuclear localization, no function for GLOIN261 could be hypothesized due to lack of interacting plant proteins. Its correct protein interactors may have been missed because of their absence in the cDNA library, because this library did not contain material from mycorrhized roots, but GLOIN261 may also associate with other biomolecules, such as DNA and RNA. Subjecting GLOIN261 to chromatin immunoprecipitation (ChIP) or cross-linked RNA IP (CLIP) experiments may shed more light on its host target molecules.

Previously, RiSP749 has been predicted to be a large, secreted protein without differential expression in three different host plants, *i.e.*, *M. truncatula, Brachypodium distachyon* (stiff brome), and *Lunularia cruciata* (crescent-cup liverwort) (Kamel et al., 2017). In contrast, we found *RiSP749* to be expressed in tomato mycorrhized root regions and specifically enriched in arbuscule-containing root segments at 6 weeks, highlighting the importance of studying effectors in a host plant of interest (Lanfranco et al., 2018; Zeng et al., 2018). Just like for GLOIN261, ectopic expression of *RiSP749* did not affect mycorrhization and growth which might be because of the poor ectopic expression levels we obtained. Nevertheless, it interacted with the serine/arginine-rich (SR) splicing factor RSZ22 (LOC050) (Barta et al., 2010) inside the nucleus, which was also highly expressed at later stages of symbiosis. Unfortunately, no *LOC050* silencing lines could be generated, hinting at an essential role in plant growth. Both RiSP749 and LOC050 exhibit RNA-binding motifs and the homology of RiSP749 to small nuclear ribonucleoproteins strongly suggests a role in mRNA splicing for example of specific genes involved in metabolic processes important for arbuscule-containing cells. Detailed investigation of the RNA targets of both LOC050 and RiSP749 in these cells by tissue-specific RNA-sequencing approaches of transgenic lines or CLIP experiments will help to validate this hypothesis. Certainly because it recently has been demonstrated that other *R. irregularis* effectors can also interact with specific SR splicing factors to regulate alternative splicing in potato (Betz et al., 2023), and certain effectors secreted by the phytopathogen *Phytophthora* can reprogram their host by modulating alternative splicing of host mRNAs in tomato (Huang et al., 2020).

In conclusion, following our pipeline (Aparicio Chacón et al., 2023), we were able to select, identify and characterize four unknown *R. irregularis* nuclear-localized effector proteins with different modes of action in tomato. Moreover, we used effector interactomics to identify unknown host plant genes involved in AM, reflecting how effectors can be used as fishing strategies to elucidate new AM players. However, our study revealed the drawback of ectopic expression in functional characterization, requiring loss-of-function studies, either by fungal mutagenesis or virus- and host-induced gene silencing, for a final validation of their function. Experiments that consider the combinatorial influence of effectors, as well as the likelihood that effectors also target host plant DNA and/or RNA in different plant hosts, will help to unravel the complex communication between mutualistic fungi and their hosts.

## Data availability statement

The datasets presented in this study can be found in online repositories. The names of the repository/repositories and accession number(s) can be found in the article/**Supplementary Material**. The sequencing data underlying this article are available in ArrayExpress collection in BioStudies, and can be accessed with E-MTAB-13691.

## Author contributions

MA: Writing – original draft, Writing – review & editing, Investigation, Visualization, Formal analysis. SL: Writing – original draft, Investigation. VD: Writing – original draft, Investigation, LR: Writing – original draft, Investigation. TL: Writing – original draft, Investigation. NS: Writing – original draft, Investigation. AD: Writing – original draft, Investigation. EC: Writing – original draft, Resources. AG: Writing – original draft, Resources. SG: Writing – original draft, Writing – review & editing, Conceptualization, Project administration, Supervision. JVD: Writing-original draft, Writing – review & editing, Conceptualization, Project administration, Supervision.
