# Supplementary material for: Exploring the potential role of four Rhizophagus irregularis nuclear effectors: opportunities and technical limitations
Source: Front Plant Sci. 2024 Apr 24;15:1384496. doi: 10.3389/fpls.2024.1384496 (PMC11085264; doi:10.3389/fpls.2024.1384496)
Supplement: Supplementary file 1 [file DataSheet_1.docx]

**SUPPLEMENTAL INFORMATION**

**GLOIN707 (GLOIN_2v1591707; RirG040740; GBC29935.2)**
MYKLNIIIIFFLCIAAIFTNAAPLNTVPQNPSSSLTARSALNLNSDASAIYHAKKRRGIIIPELDLSDSDTIRPKIIKNRMIKRNPINYNAANHSTKDAVSQEKRAQDKKRAQKKERRSIVWKVDENILFKRNEK

**GLOIN781 (GLOIN_2v1603781; jgi.p|Gloin1|349745; GBC42057.1)**

MKKIISLLILIVLLISSLSVIESRLDQDEEPFMEFDDNMMLPLIISAGENINEYTISSGGHHHHPKY**PKH**PKHPKHPKFPGPKRAFAYFPSPPDITKGVVVFWETSKNNTLVYGQFSKGFVEGEEDNYSFKVYKGDQELVDLKPKDDDLDRILKINPNGSTDLFLFVFNDTLISGVGILDTDLVISTSDSLIGKDRIKPLTCW

**GLOIN261 (GLOIN_2v1478261; RirG045970; GBC25372.1)**

MNRLHILIFILFTFLFVTAFSEEDLIPVKQLTANLLKIRKVGHNKLIAEVTWDGTFERDDEPVKTKFRCFSDAVTVKGPKHGVFGDRKVNFEIKVHKKNVKVKCRYGTKDISSFKNVFYFRT
**RiSP749 (Gloin_2v1708442 ; RirG117440 ; GBC20232.1)**

MWYAKNFLQFLILIIQEMWYAKKYDPLQAGSIDGTDTVPHDHGILRAQNSNYVPPSDKDDVTSDPFHTIFVGRLNPDTTEETLTQVFEKFGTIKKIRLVRNIVTGDSRGYAFIEFTHERSCQEAYRYAYKMTIDGRQILVDYERSRIMEGWIPRRMGGGFAGRKESGQLRFGARDRPFKRPLNIAVNQYMPEILPDQRFDDCWRHNANRRGTNSSSSATQNYRSIFQYSKDLHFNRQGKSSITGISNYNPRNSPDEERFTYNKRNESPSRFHHEKSIDSFSSRHRVHTNEFHQAENKYNQEKKFRRYSKSRSRSPPRYRHNNREKSEYRRSRSRDKNQDRYIHSKEKYKSHERRRNNEHRR

**Supplementary Figure 1.** Amino acid sequences of putative effector proteins. The SP is highlighted in brown, while the NLS is highlighted in yellow. Effector CDSs (with SP) were cloned from mycorrhized tomato cDNA and the subsequent translated protein sequence was blasted against the latest *R. irregularis* proteome RIR17. While no mismatches were identified between the annotated and the cloned translated protein sequence of GLOIN707, GLOIN261, and RiSP749, the effector protein GLOIN781 lacked the PKH amino acids located in its NLS at position 68 to 70 (Underlined). Between brackets the following information is displayed: the first effector ID corresponds to the effectome from Tisserant & Brachmann., 2016; the second ID matches the secretome of Zeng *et al.*, 2018; while the last ID refers to the RIR17 proteome annotation (Maeda *et al.*, 2018).


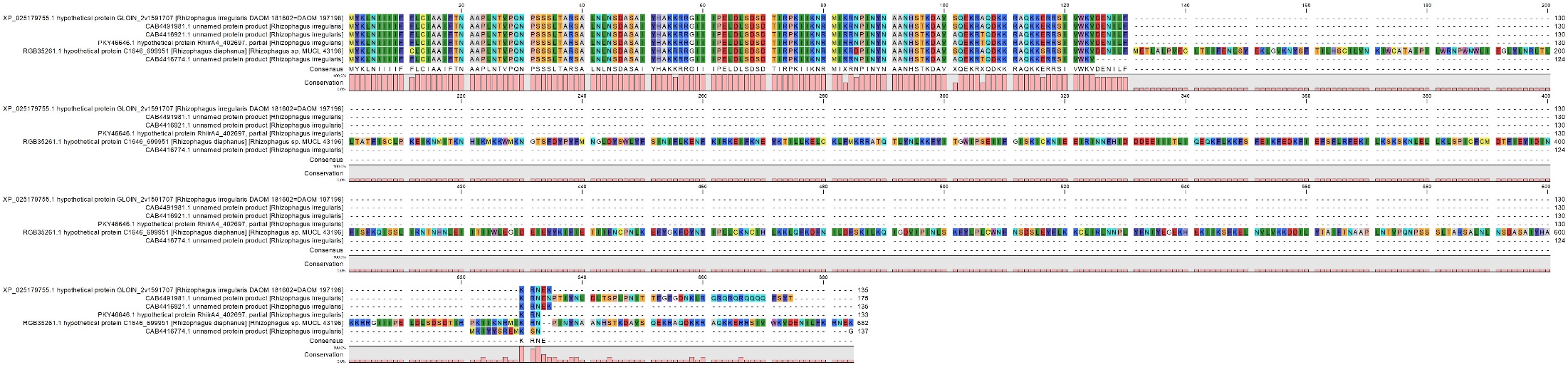


NLS

GLOIN781

NLS

SP

SP

GLOIN707

**(a)**

SP


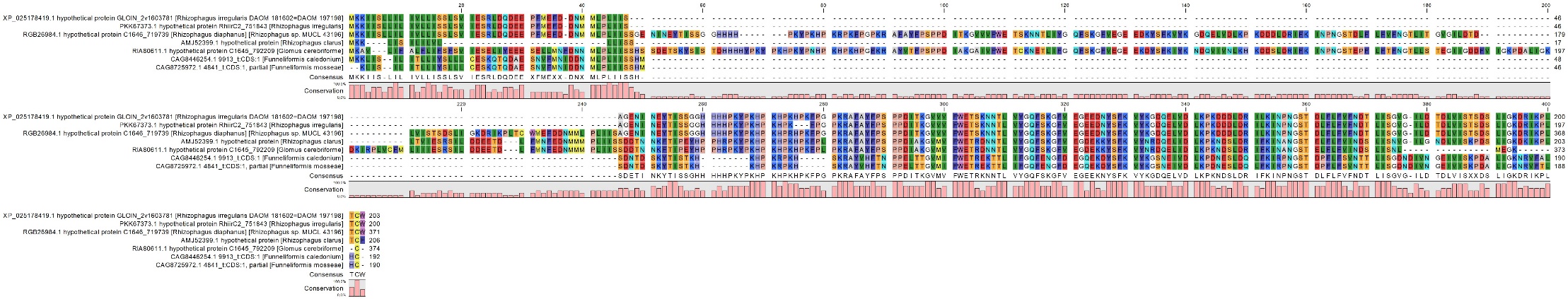


NLS


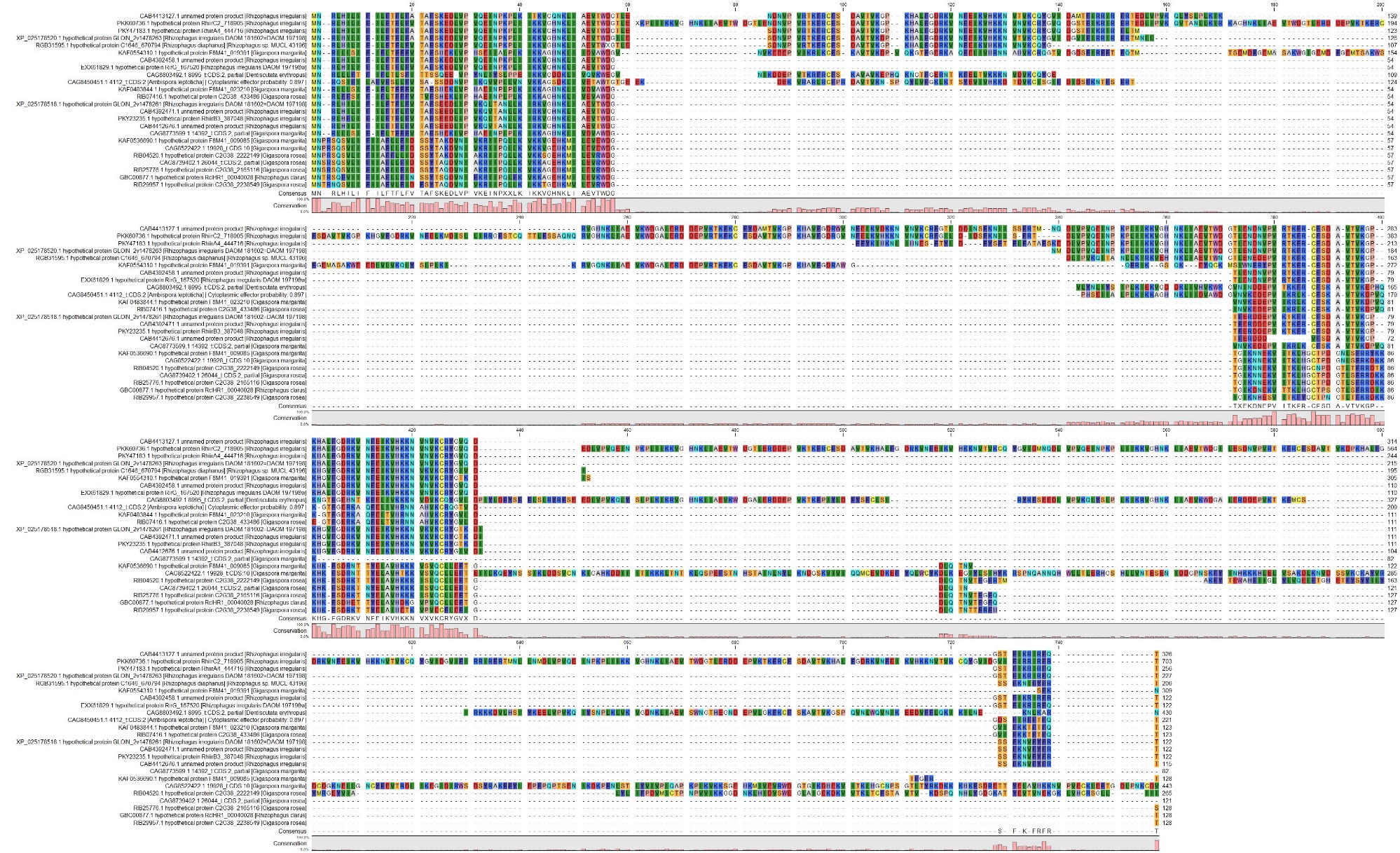


SP

NLS

GLOIN261

RiSP749

**
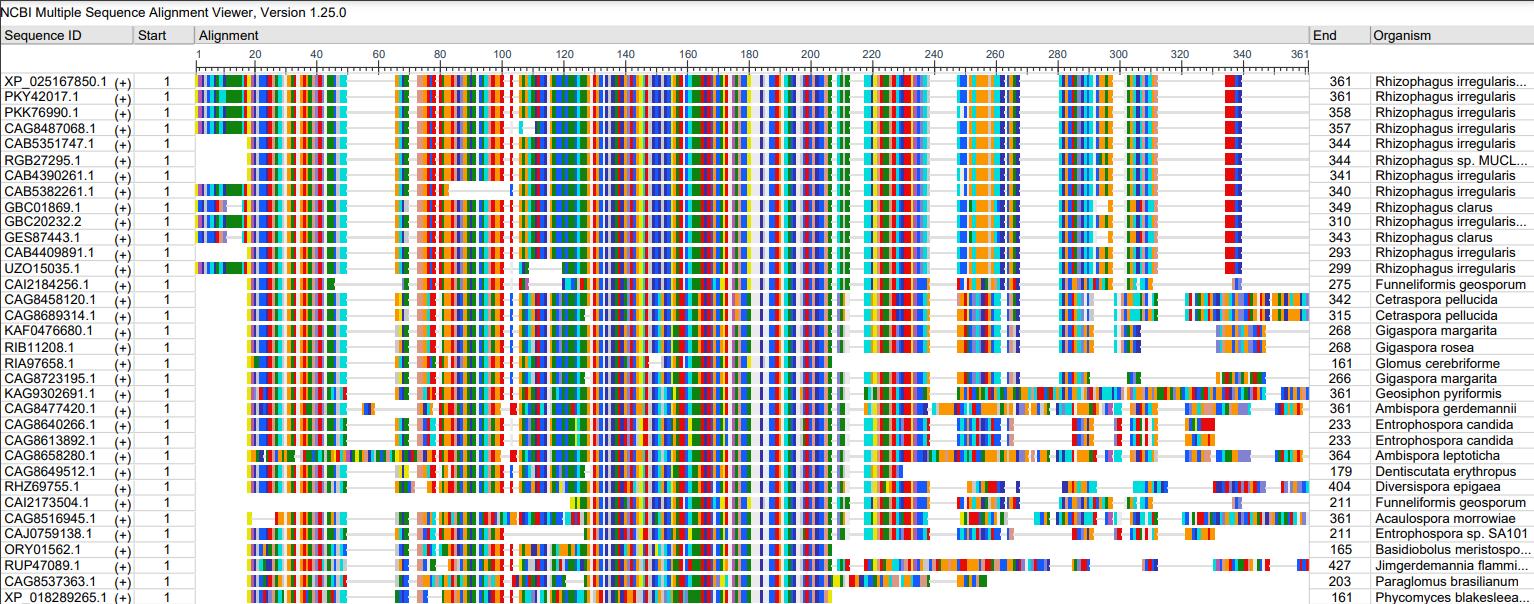
**

NLS

SP

NLS

**
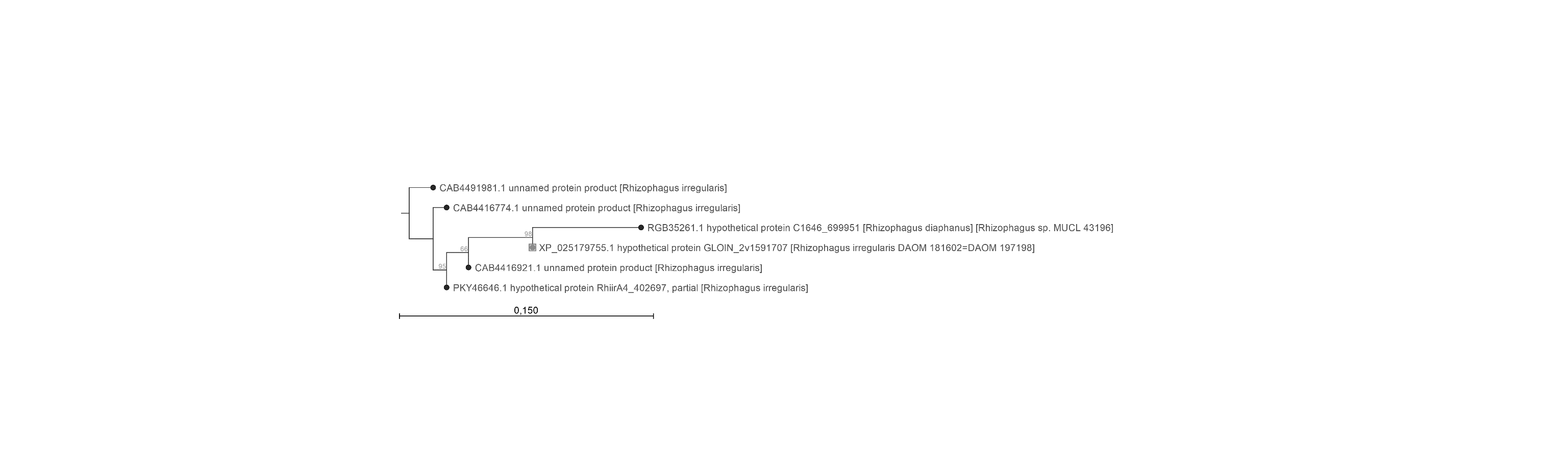
**


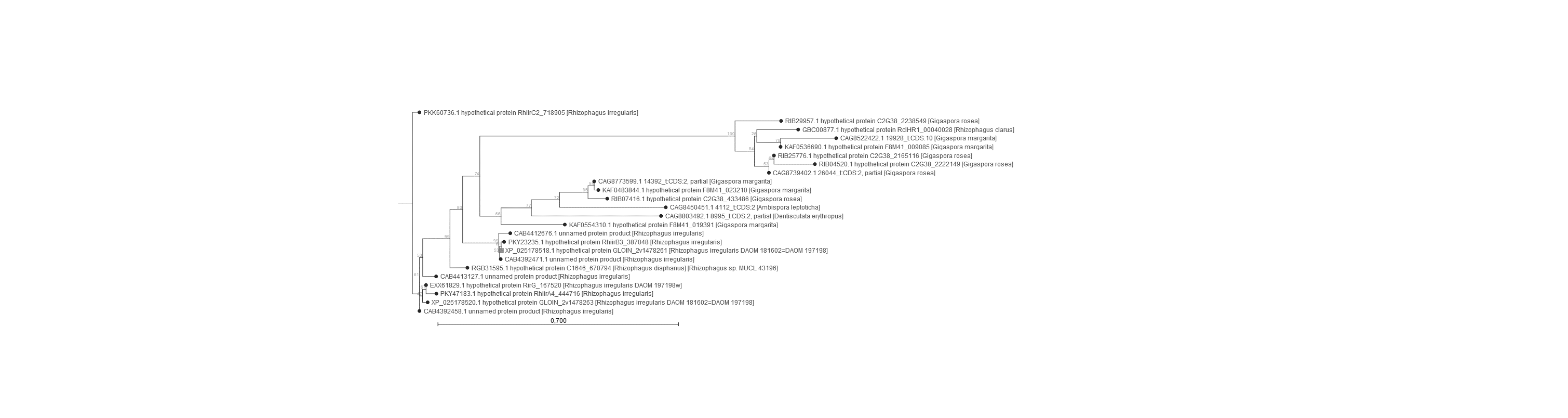

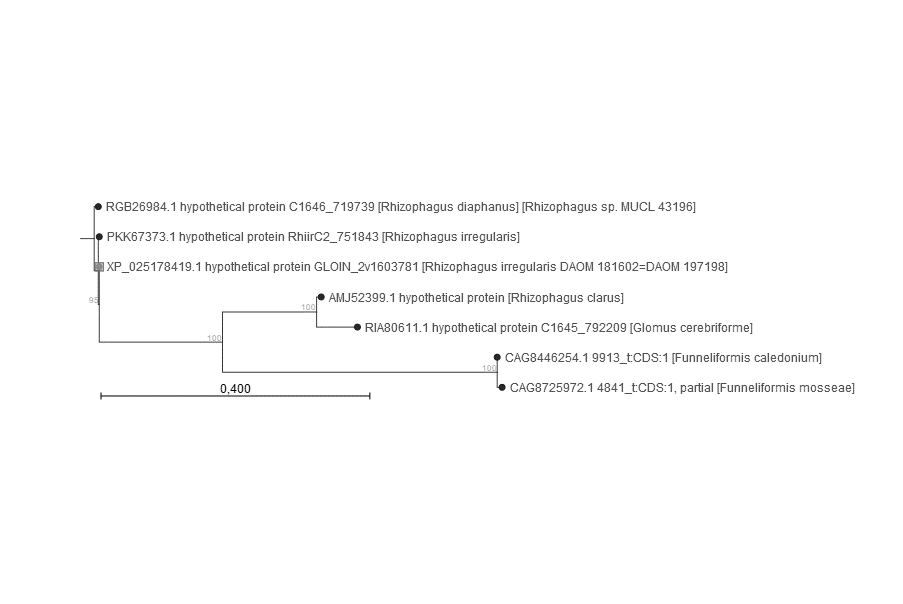


**(b)**

GLOIN781

GLOIN707

GLOIN261

RiSP749

**
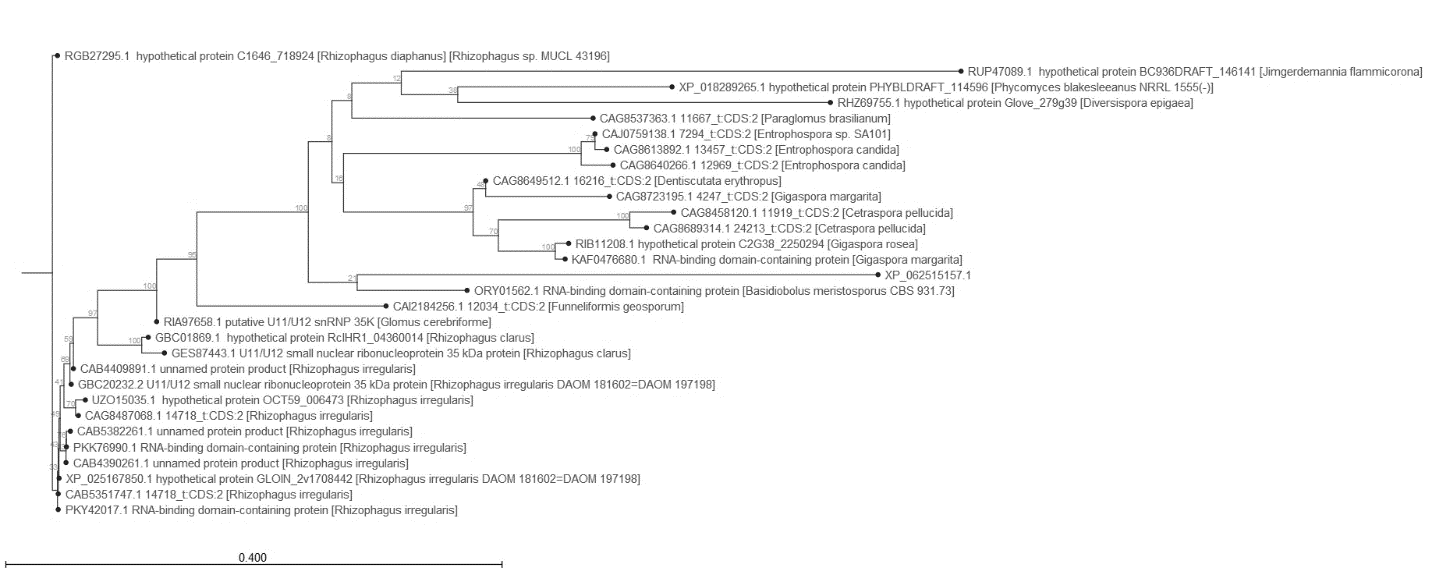
**

**Supplementary Figure 2.** Homologous proteins of GLOIN707, GLOIN781, GLOIN261, and RiSP749 in other fungal species. (a) Protein alignment with indications of motifs in rectangles of selected proteins for (b) phylogenetic relationship among nuclear effector-like homologous proteins inferred by applying the Maximum Likelihood method with 1000 bootstrap replicates using CLC Workbench 8.1 software. GLOIN707, GLOIN781, GLOIN261, and RiSP749 effectors are highlighted in yellow.

**SL296**

**
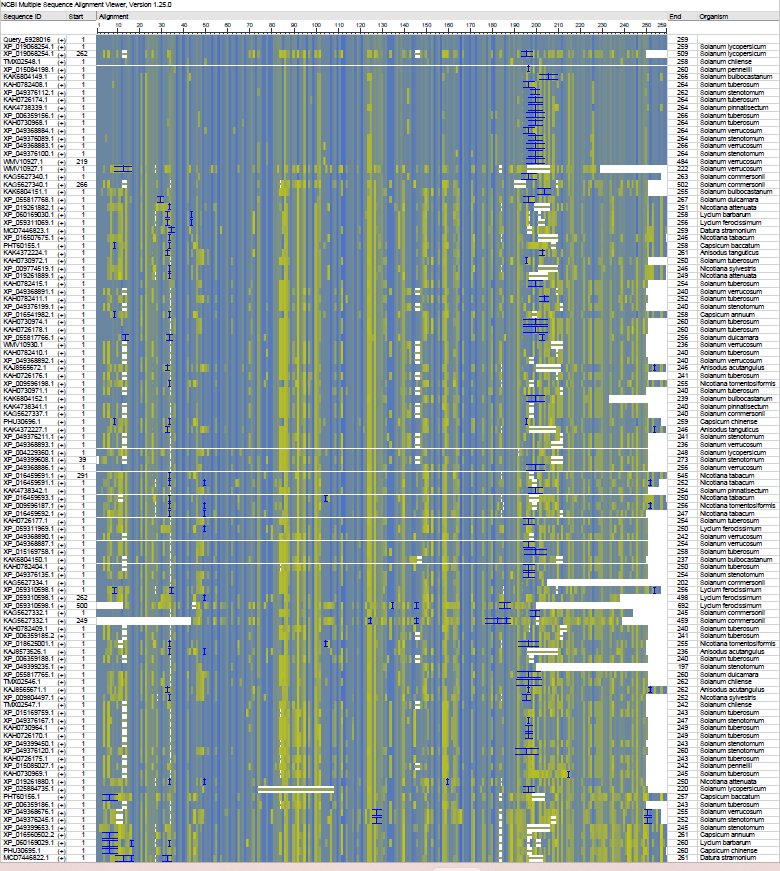
**

3x Cys1

**SlGLY**

**
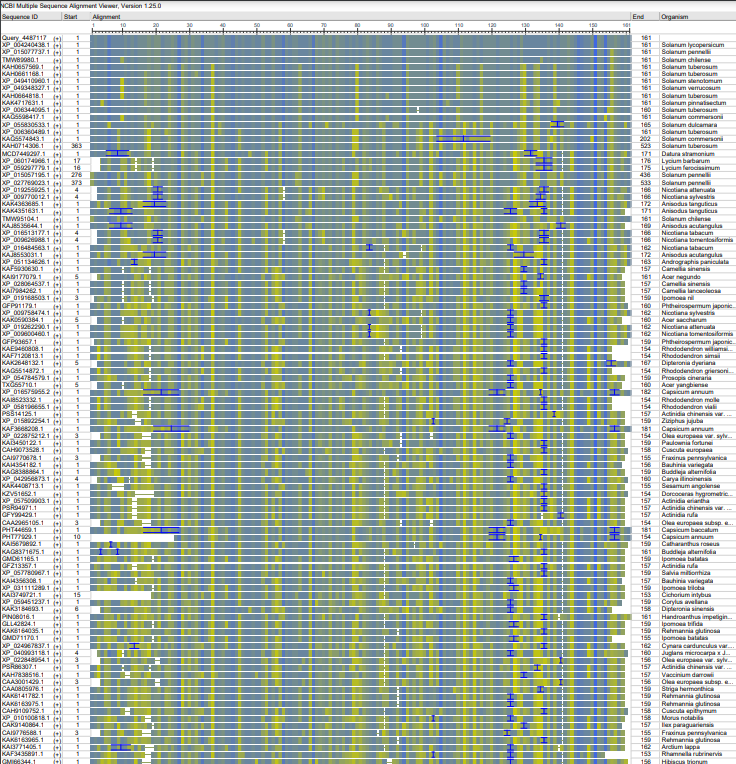
**

**Gly**

**Supplementary Figure 3.** Homologous proteins of Sl296 and SlGLY obtained with a broad BlastP search against non-redundant protein databases in NCBI. Sl296 homologous proteins displaying >57% identity (e-value 2e – 95) with indication of Cys1, Cysteine/Histidine-rich C1 domain; and SlGLY >55% identity (e-value 2e – 47) with indication of Gly, glyoxalase domain, were retained and a multiple alignment was conducted using the NCBI MSA Viewer 1.25 online tool. Amino acid residues are highlighted in agreement with the BLOSUM80 matrix to display the degree of match of residues relative to each alignment position/column. The coloring in the column shows the match score to the residue of the query: blue represents a better match while green represents a worse match.


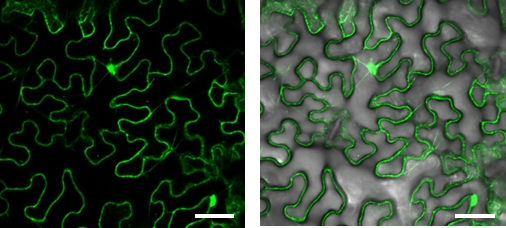


GFP

GFP merged with BF

**(a)**


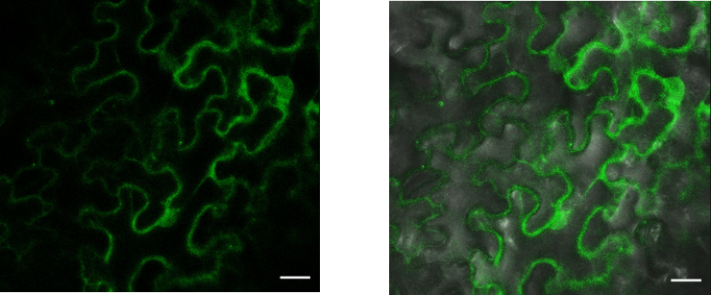

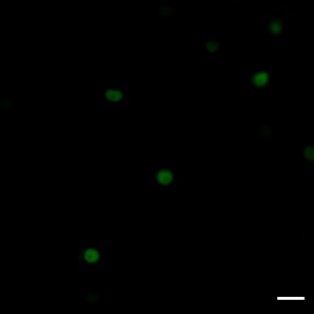

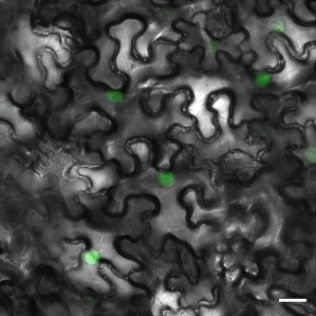

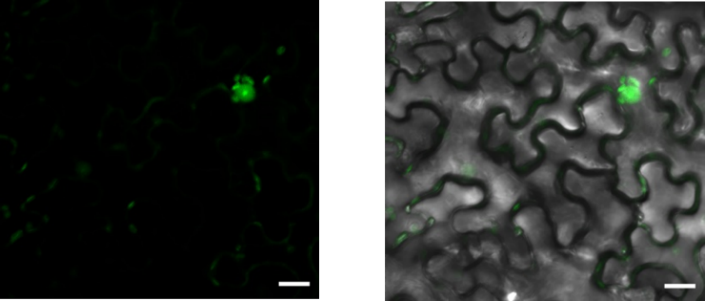

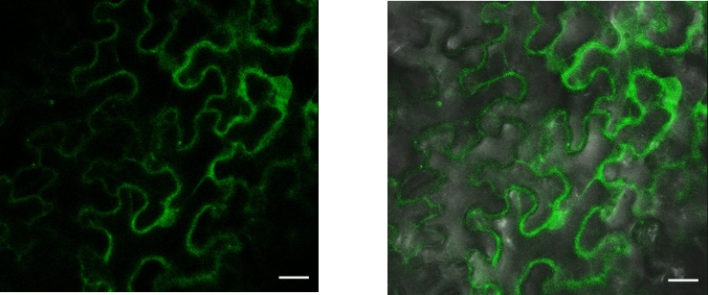

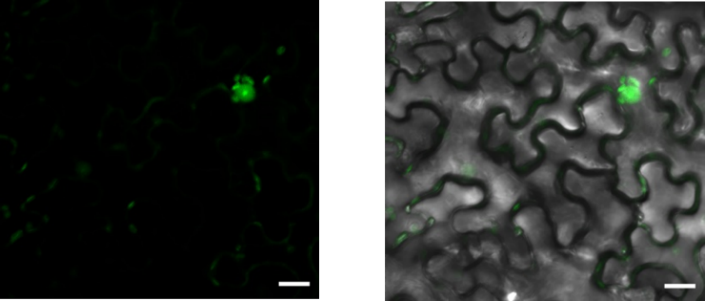

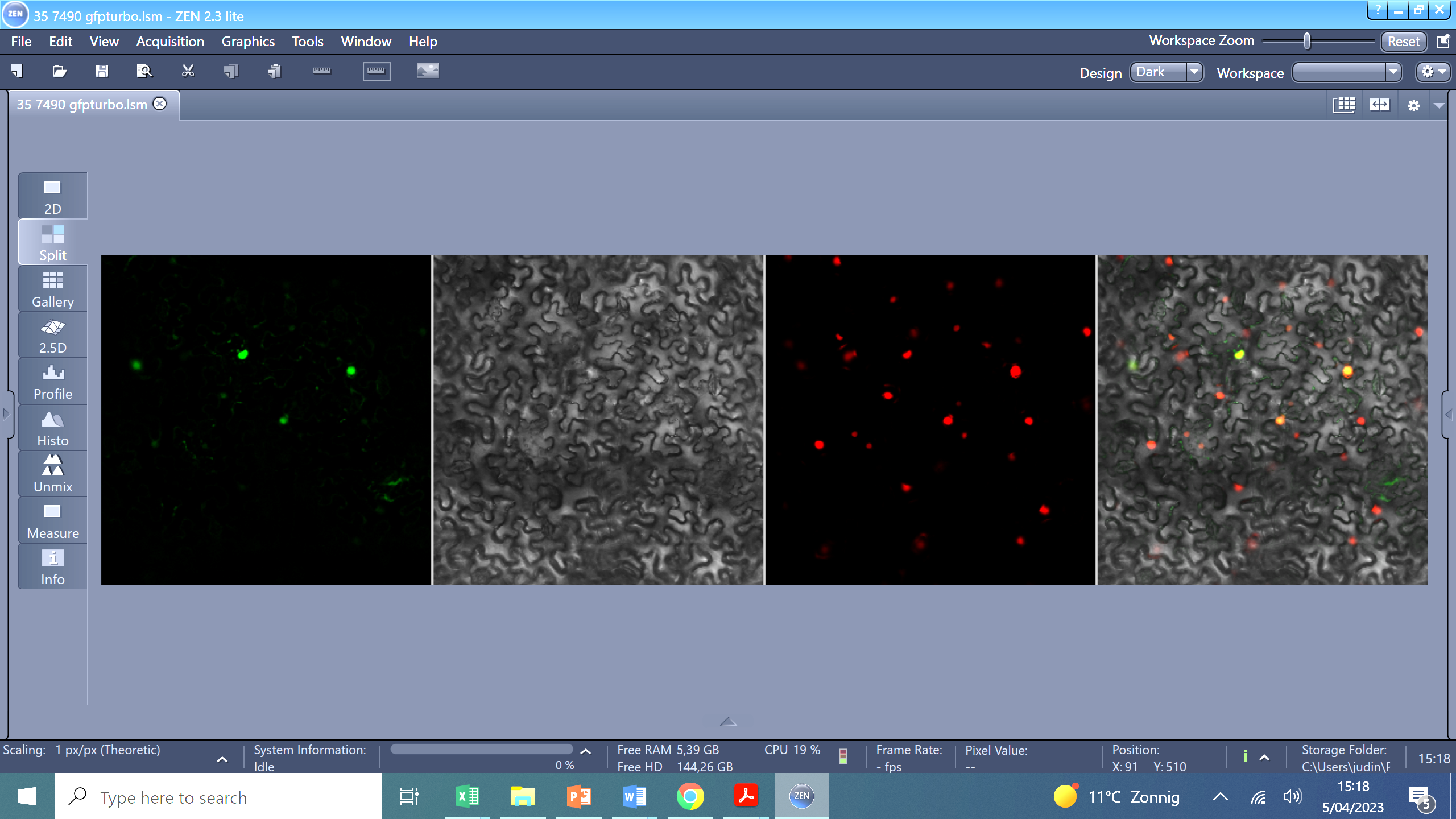


**(b)**

**(c)**

**(d)**

**
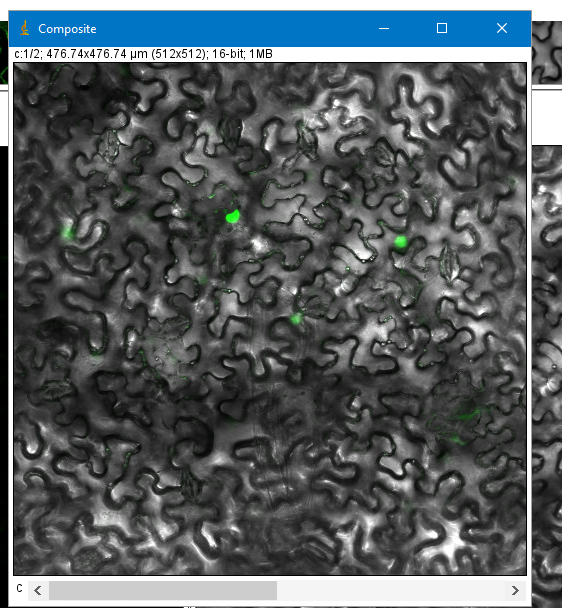
**

**(e)**

**Supplementary Figure 4.** Localization of free GFP (a), the C-terminal GFP fusion proteins of GLOIN707 (b) and GLOIN781 (c), the N-terminal GFP fusion protein of RiSP749 (d) and the C-terminal GFPTurbo protein of RiSP749 (e) in *N. benthamiana* leaves. BF: bright field. Scale bars are 20 µM in (b-d), 50 µM in (a,e).


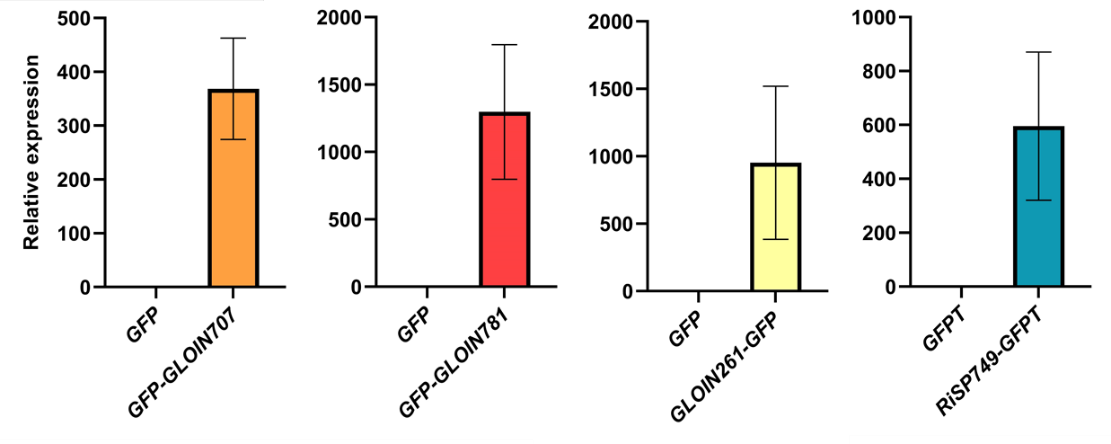
 **(a)**

***GLOIN707***

***GLOIN781***

***GLOIN261***

***RiSP749***

**(b)**


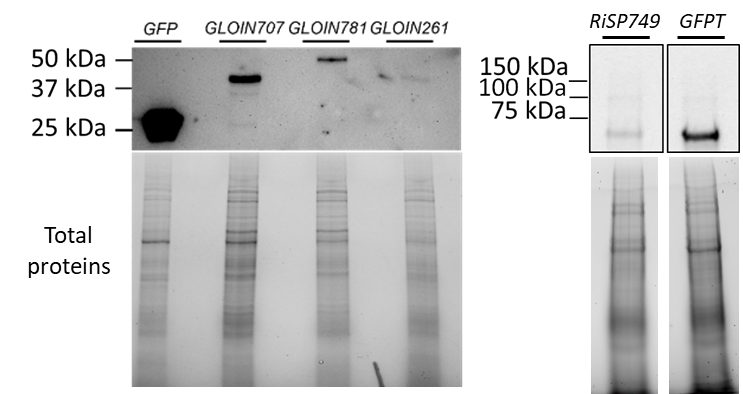

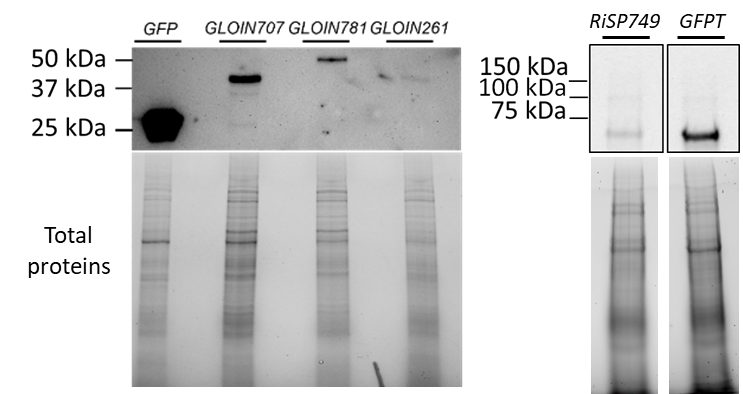

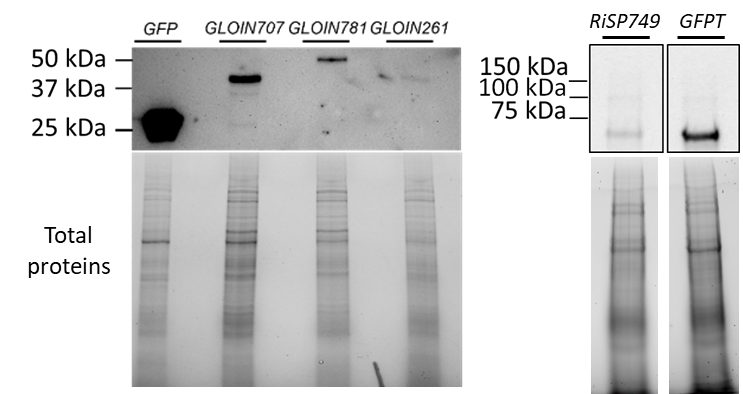


**Supplementary Figure 5.** Effector expression in *M. truncatula* transgenic roots. (a) Effector transcript levels in *M. truncatula* roots inoculated with *A. rhizogenes* carrying the vectors *35Sp:GFP-GLOIN707*, *35Sp:GFP-GLOIN781*, *35Sp:GLOIN261-GFP*, *35Sp:RiSP749-GFPTurbo* (*RiSP749-GFPT*), *35Sp:GFP*, or *35Sp:GFPTurbo* (*GFPT*) measured by qRT-PCR at 4 weeks post inoculation. Transcript levels were normalized with *MtGAPDH.* Values are means ± SEM of three independent biological repeats. (b) Effector fusion proteins detected by Western Blot using anti-GFP monoclonal antibodies. Molecular mass of fusion proteins: GFP, 26.9 kDa; GFP-GLOIN707, 40 kDa; GFP-GLOIN781, 47 kDa; GLOIN261-GFP, 39 kDa, RiSP749-GFPT, 102 kDa; and GFPTurbo (GFPT), 68 kDa. Total amounts of proteins are shown after separation on stain-free polyacrylamide gels and were visualized under stain-free conditions using the ChemiDoc imaging system (Bio-Rad). The amount of total protein loaded per well in this western blot corresponds to 25 ng per sample. Each sample contains a pool of three to six composite root systems in which different levels of effector protein expression is expected

**(a)**

***GFPT***

***RiSP749-GFPT***

***GLOIN261***

***GLOIN781***

***GLOIN707***

***GFP***


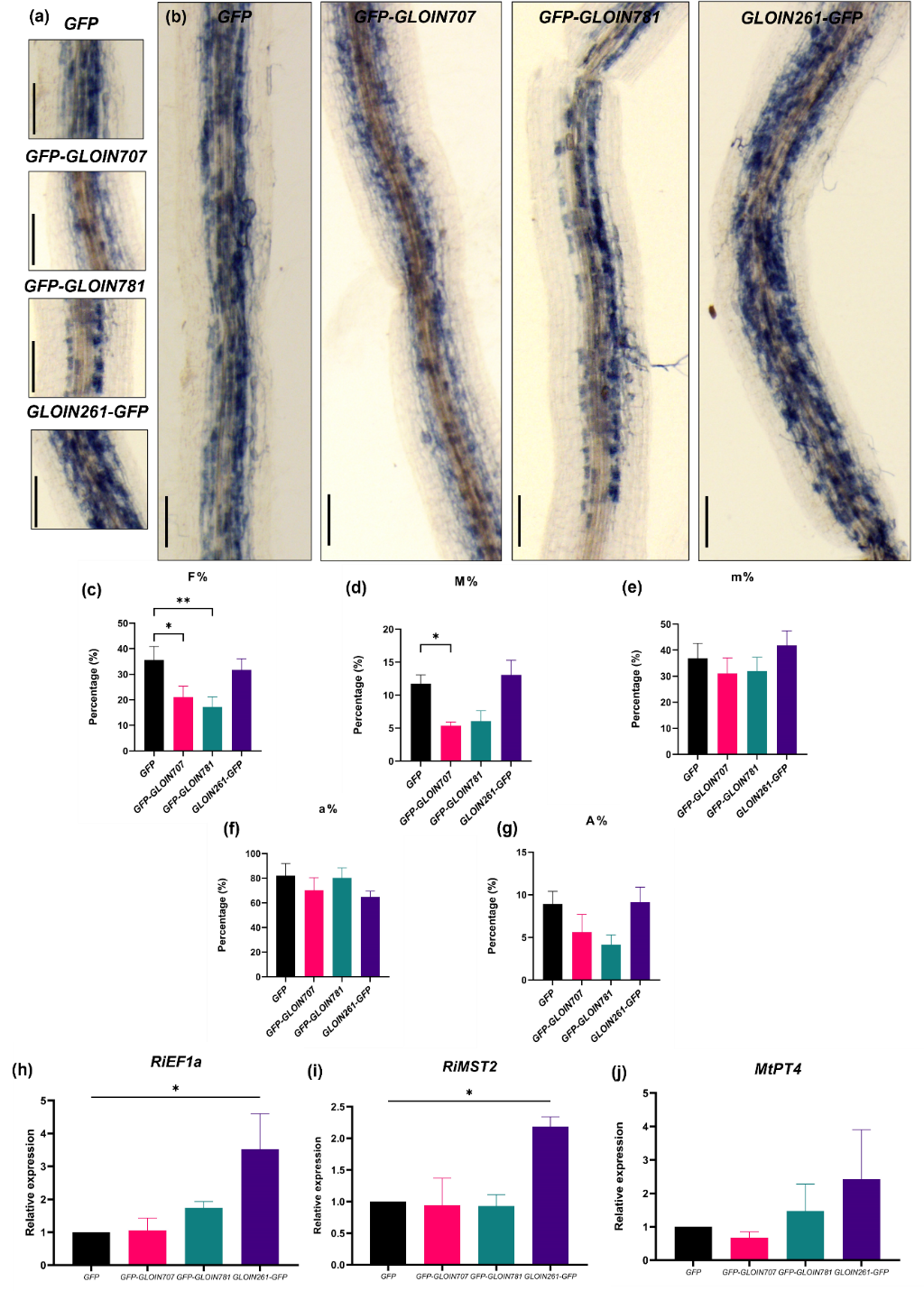


**(b)**

**
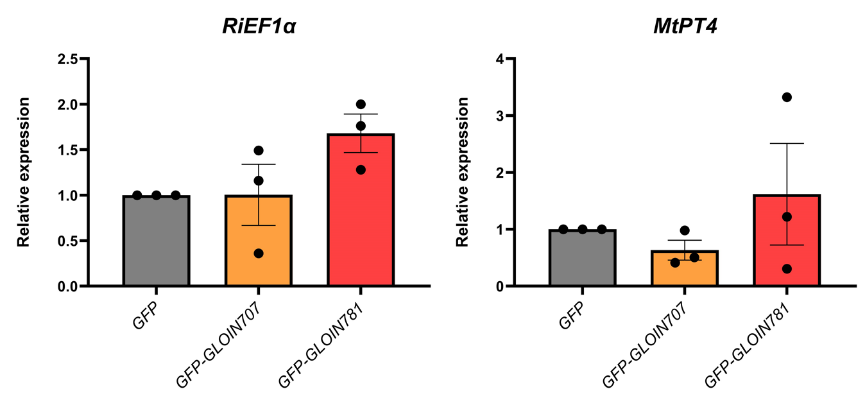
**

**Supplementary Figure 6.** Mycorrhized *M. truncatula* roots ectopically expressing the effector fusions. (a) Representative bright-field images of ink-stained 4-week-old mycorrhized M. truncatula roots expressing *GFP* (control), *GFP-GLOIN707*, *GFP-GLOIN781*, *GLOIN261-GFP*, *GFPTurbo* (*GFPT*, control) and *RiSP749-GFPT*. Bars = 100 µM. (b) Expression analysis of *RiEF1α* and *MtPT4*. Transcript level normalization was conducted using *MtGAPDH.* Values are represented as a mean ± SEM of three independent biological repeats.

**(a)**

**
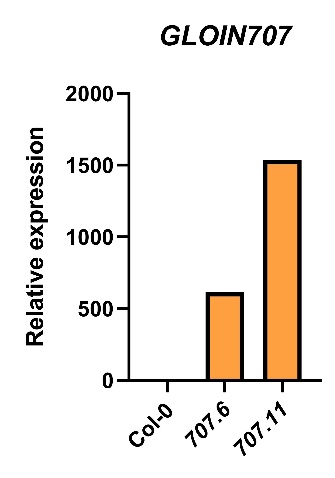

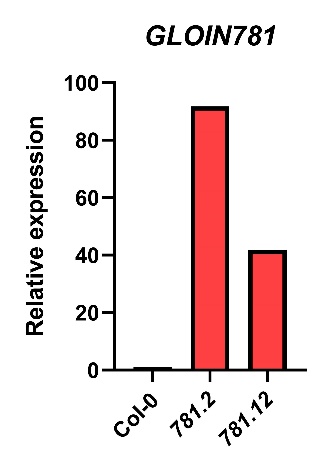

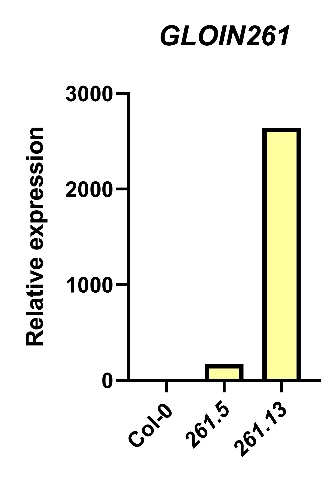

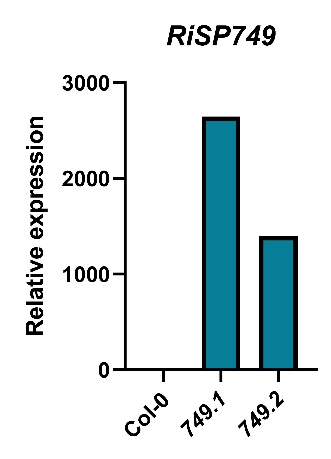
**

***RiSP749***

***GLOIN261***

***GLOIN781***

***GLOIN707***

**(b)**

**
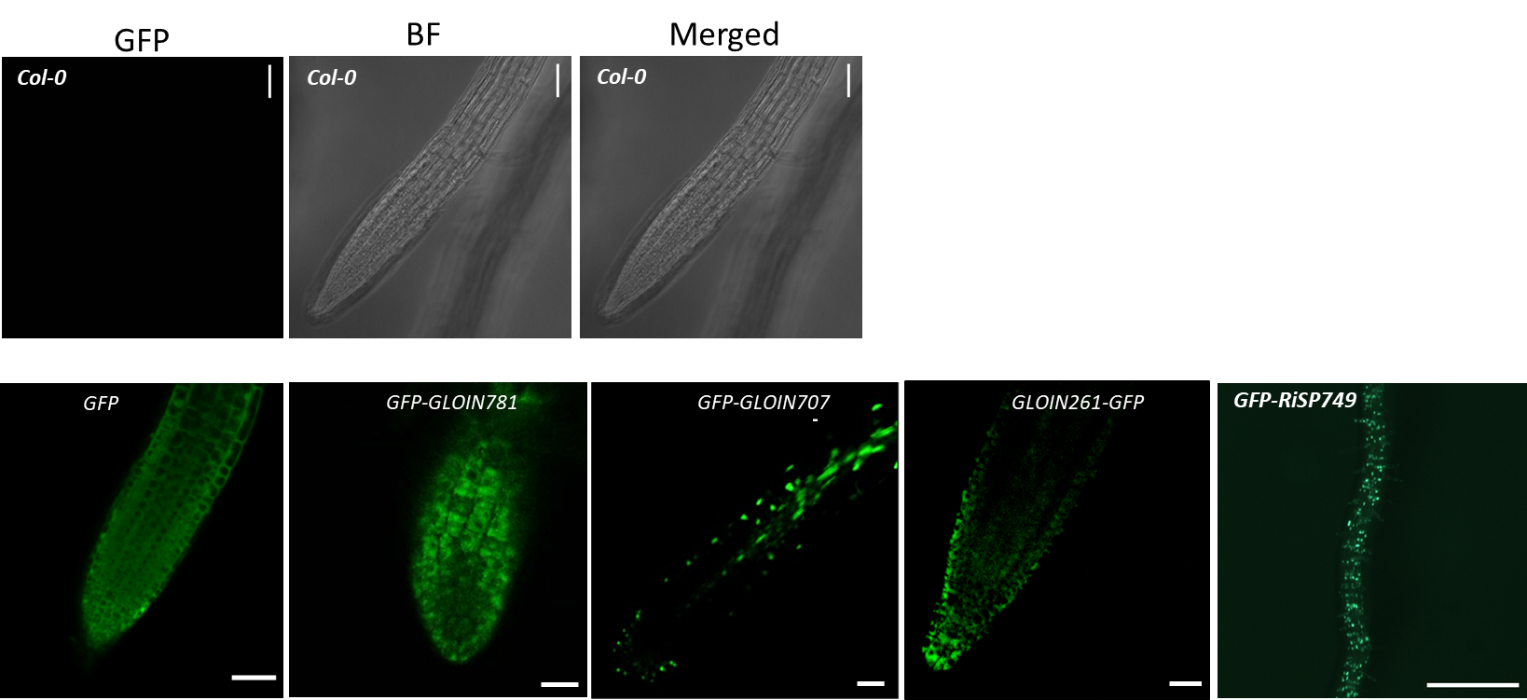
**

**Supplementary Figure 7.** (a) Expression analysis confirming overexpression of the respective effector fusions in roots of Arabidopsis transgenic T3 lines expressing the 35Sp:*GFP-GLOIN707* (GLOIN707.6 and GLOIN707.11), 35Sp:*GFP-GLOIN781* (GLOIN781.2 and GLOIN781.12), 35Sp:*GLOIN261-GFP* (GLOIN261.5 and GLOIN261.13), and 35Sp:GFP-*RiSP749* (RiSP749.1 and RiSP749.2) with their respective WT Col-0 control plants at 14 DAS. Transcript level were normalized using *AtACTIN* and *AtTUBULIN* and relatively compared to WT control Col-0 roots. (b) Top, representative confocal laser-scanning microscopy images of 14 days old root tips of *A. thaliana* Col-0. BF = bright field. Bottom, representative confocal laser-scanning microscopy images of GFP fluorescence of 14 days old root tips of *A. thaliana* expressing GFP (bar, 50 µm) and *GFP-GLOIN781, GFP-GLOIN707, and GLOIN261-GFP* (bars, 20 µm). For *GFP-RiSP749* a fluorescence microscopy image of 8 days old roots of *A. thaliana* expressing *GFP-RiSP749* is shown (bar, 1 mm).


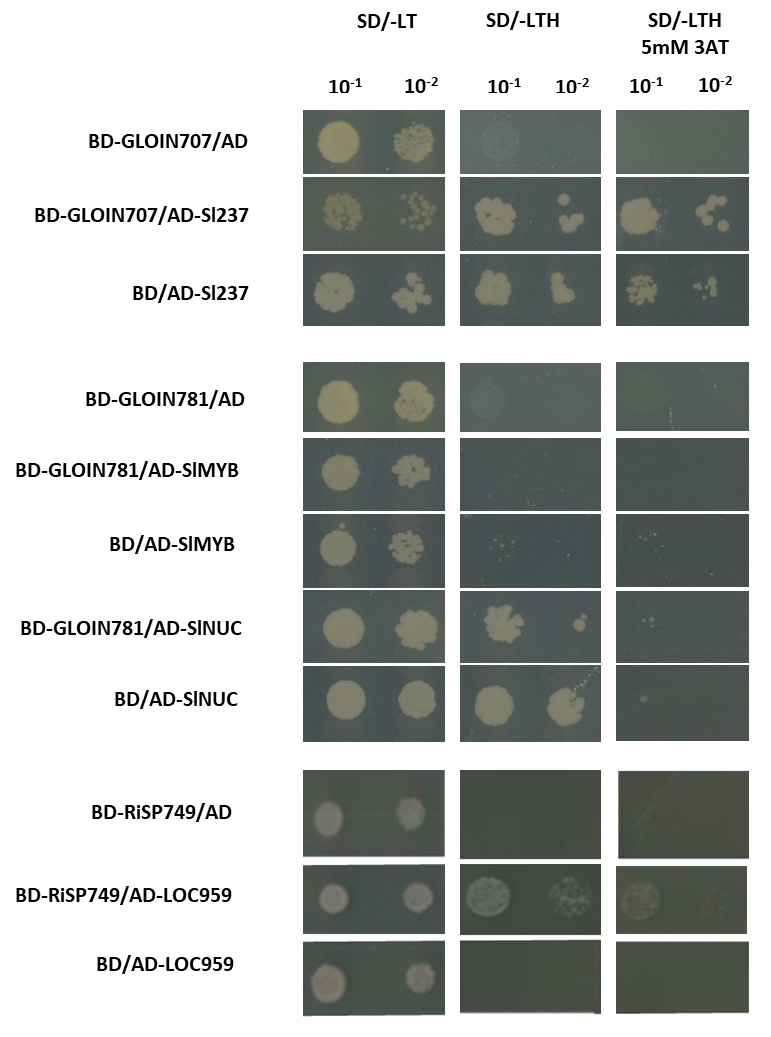


**Supplementary Figure 8.** Binary Y2H assays using the effector as baits in PGBKT7 vector (BD-) and tomato prey protein in PGADT7 (AD-). Constructs were cotransformed in Pj69-4α *S. cerevisiae* cells, after which they were serially diluted (10× and 100×) and grown on SD/-LT control medium, SD/-LTH and on SD/-LTH selective medium supplemented with 5 mM of 3-AT for 3 days at 30°C. Interaction of GLOIN707 (BD-GLOIN707) with the autoactive tomato protein Sl237 (AD-Sl237) was confirmed in selective medium supplemented with 5 mM 3-AT, whereas no interaction between GLOIN781 (BD-GLOIN781) and SlMYC and SlNUC (AD-SlMYB and AD-SlNUC) proteins, while RiSP749 (BD-RiSP749) weakly interacted with LOC959 (AD-LOC959).


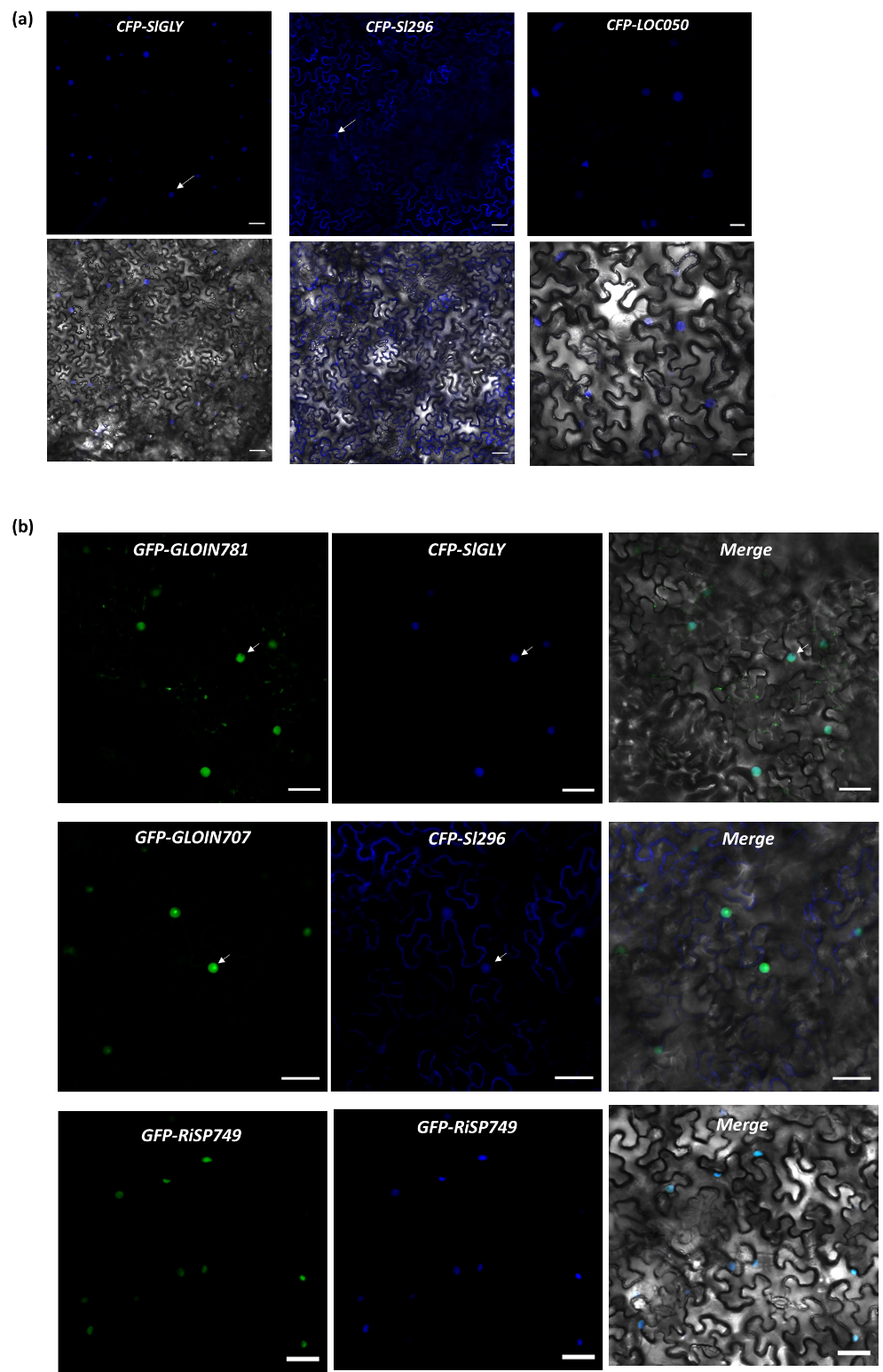


CFP merged

with BF

CFP

GFP/CFP merged

with BF

CFP

GFP

**
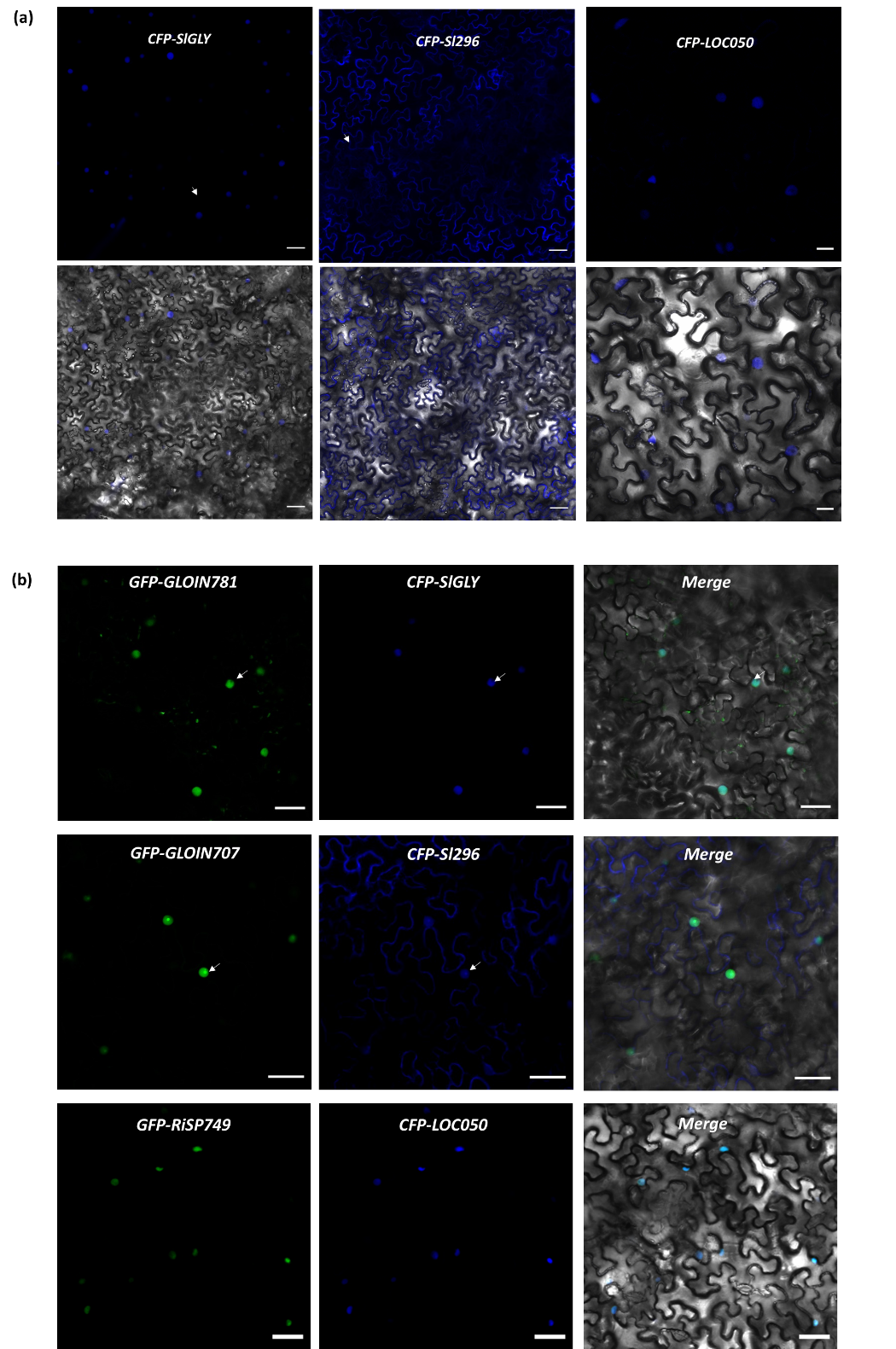
**

**Supplementary Figure 9.** Subcellular localization of SlGLY, Sl296 and LOC050 CFP fusion proteins in epidermal *N. benthamiana* leaf cells. (a) Single and (b) coinfiltrations of CFP fusion proteins of SlGLY, Sl296 and LOC050, and GFP fusion proteins of GLOIN707, GLOIN781 and RiSP749. Scale bars = 50 (a) and 20 µM (b). BF: bright field. White arrows point to nucleus.

**Supplementary Figure 10.** Relative expression in mycorrhized composite tomato *SlGLY* and *Sl296* RNAi lines at 4 weeks post inoculation. Reduced expression of (a) *SlGLY* and (b) *Sl296* in respective RNAi lines. *SlGAPDH* was used as reference gene for normalization and compared to mycorrhized lines containing the empty hairpin construct (EV). Data are means ± SEM (n = 3-6; *P < 0.05, ***P < 0.001; Student’s t-test) and three independent biological experiments are represented. For each biological repeat, 3 to 6 independent RNAi or EV root systems were pooled for RNA extraction. (c) No difference in expression of *RiEF1α* in *SlGLY* and *Sl296* RNAi lines (Student’s t-test, P > 0.05). *SlGAPDH* was used as reference gene for normalization and compared to mycorrhized lines containing the empty hairpin construct (EV). Data are means of the three biological repeats ± SEM.

**(a)**

**(b)**


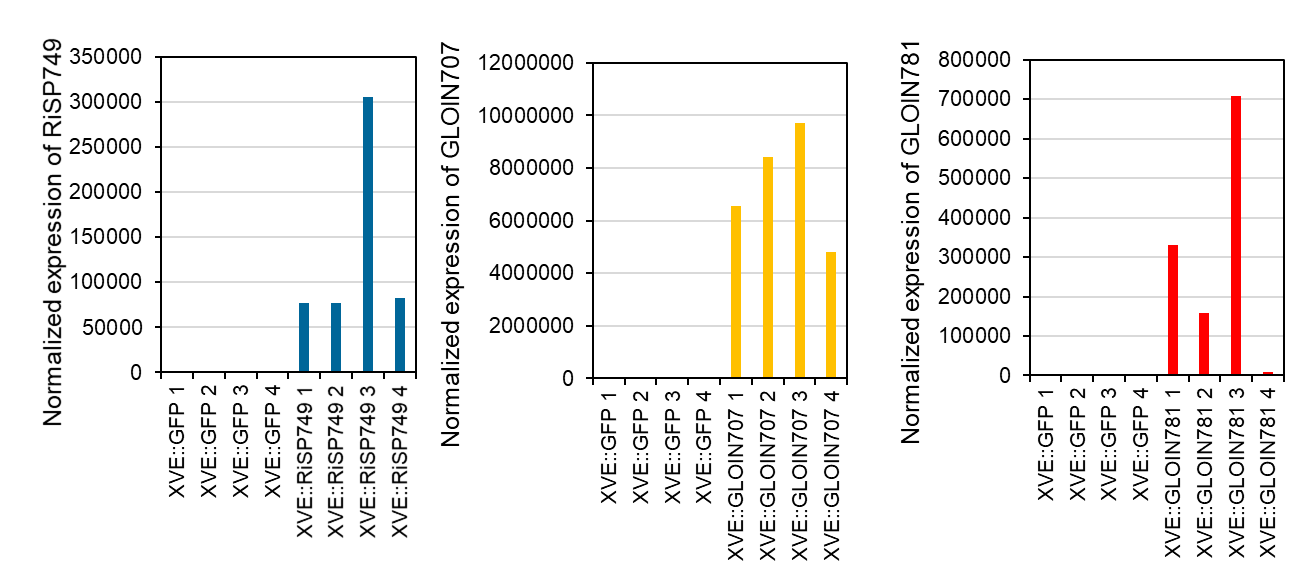


**Supplementary Figure 11.** Normalized expression of *TURBO* (a) and the respective genes (b) in *GFP*, *GLOIN707*, *GLOIN781*, and *RiSP749* in the four independent estradiol-induced hairy root cultures with *RPS5α_XVE::effector CDS-TurboID-flag* constructs used for RNA-sequencing. *SlEF1* and *SlGAPDH* were used as reference genes for normalization.


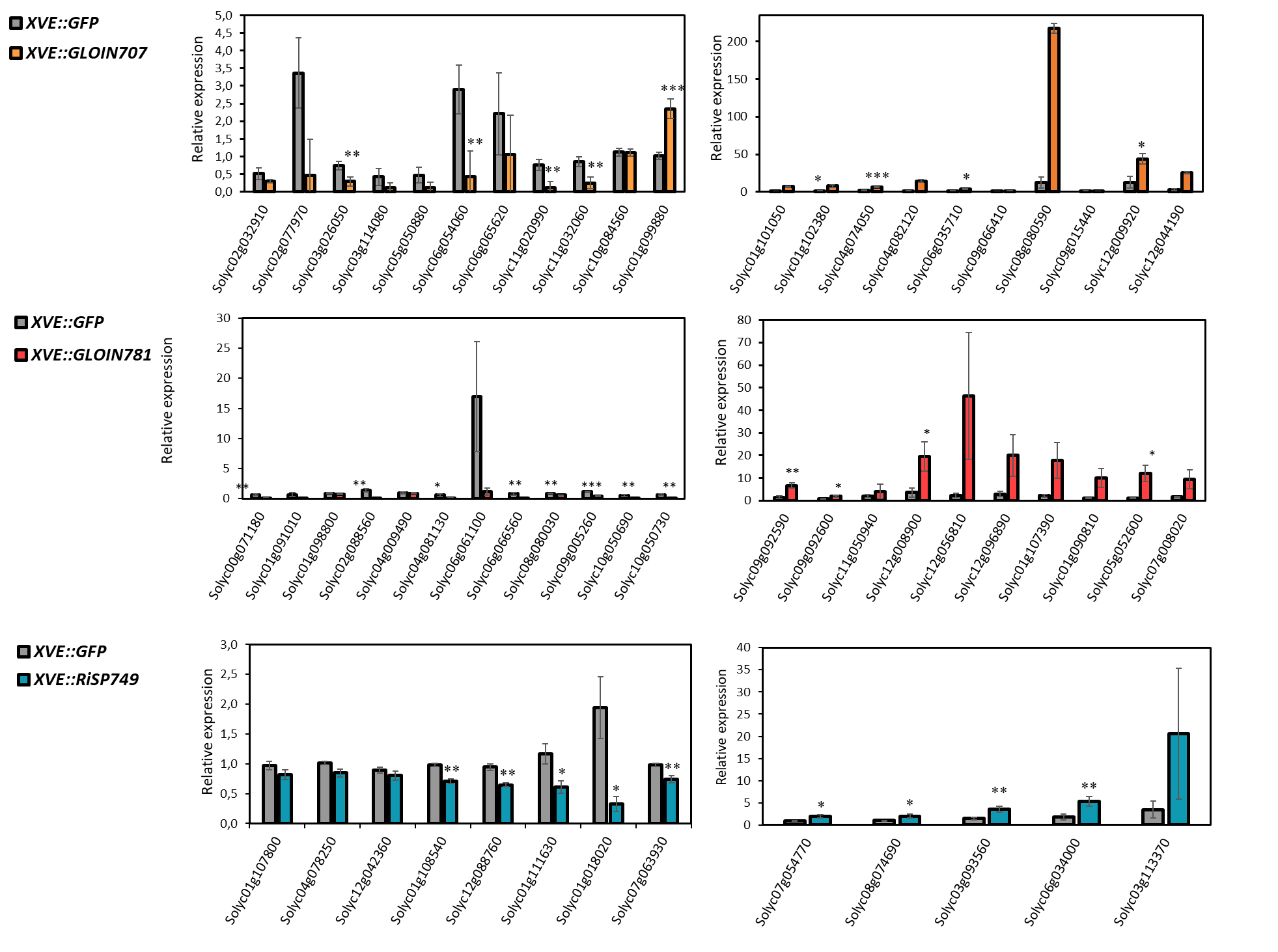


**Supplementary Figure 12.** qRT-PCR analysis of a subset of significantly up- and downregulated genes selected from the RNA-sequencing dataset. *SlEF1* and *SlGAPDH* were used as reference genes for normalization. Relative transcript levels were compared to *XVE::GFP* transgenic lines. Values are means ± SEM of four biological repeats (*n* = 4, **P* < 0.1, ***P* < 0.05, ****P* < 0.01; Student’s t-test).

**Supplementary Table 1.** *In silico* analysis of GLOIN707, GLOIN781, GLOIN261 and RiSP749 protein sequences.

*See excel file*

**Supplementary Table 2.** Oligonucleotide sequences for cloning and generation of constructs.

| GLOIN707 | PRIMER | SEQUENCE |  |  |
| --- | --- | --- | --- | --- |
| Gloin707 CDSΔSP PGGB | Gloin707_MB_F | TTTTGGTCTCAAACAATGGCACCTCTCAATACAGTACCAC |  |  |
|  | Gloin707_MB_R | TTTTGGTCTCAAGCCCTTTTCATTTCTCTTGAATAATATATTC |  |  |
| Gloin707 CDSΔSP PGGC | Gloin707_MC_F | TTTTGGTCTCAGGCTCCATGGCACCTCTCAATACAGTACCAC |  |  |
|  | Gloin707_MC_R | TTTTGGTCTCACTGATCACTTTTCATTTCTCTTGAATAATATATTC |  |  |
| Gloin707 CDSΔSP | Gloin707_AttB1 | GGGGACAAGTTTGTACAAAAAAGCAGGCTCAATGGCACCTCTCAATACAGTACCAC |  |  |
|  | Gloin707_AttB2 | ggggaccactttgtacaagaaagctgggtaTCACTTTTCATTTCTCTTGAATAATATATTC |  |  |
|  | Gloin707_AttB4 | GGGGACCACTTTGTACAAGAAAGCTGGGTTTCACTTTTCATTTCTCTTGAATAATATATTC |  |  |
| Gloin707 YST | Gloin707_YST_SP_FW | GCGCGCGAATTCATGTATAAATTAAACATTATCATAAT |  |  |
|  | Gloin707_YST_noSP_FW | GCGCGCGAATTCATGGCACCTCTCAATACAGTAC |  |  |
|  | Gloin707_YST_RV | AAGGAAAAAAGCGGCCGCCTTTTCATTTCTCTTGAATAATATATT |  |  |
|  | Gloin707_YST_SP_RV | AAGGAAAAAAGCGGCCGCAGCGTTTGTAAAAATTGCGGC |  |  |
| GLOIN781 |  |  |  |  |
| Gloin781 CDSΔSP PGGB | Gloin781_MB_F | TTTTGGTCTCAAACAATGAGATTGGATCAAGATGAAG |  |  |
|  | Gloin781_MB_R | TTTTGGTCTCAAGCCCCAACAAGTTAAAGGTTTAATCCT |  |  |
| Gloin781 CDSΔSP PGGC | Gloin781_MC_F | TTTTGGTCTCAGGCTCCATGAGATTGGATCAAGATGAAG |  |  |
|  | Gloin781_MC_R | TTTTGGTCTCACTGATCACCAACAAGTTAAAGGTTTAATC |  |  |
| Gloin781 CDSΔSP | Gloin781_AttB1 | GGGGACAAGTTTGTACAAAAAAGCAGGCTCAATGAGATTGGATCAAGATGAAG |  |  |
|  | Gloin781_AttB2 | ggggaccactttgtacaagaaagctgggtaTCACCAACAAGTTAAAGGTTTAATC |  |  |
|  | Gloin781_AttB4 | GGGGACCACTTTGTACAAGAAAGCTGGGTTTCACCAACAAGTTAAAGGTTTAATC |  |  |
| Gloin781 YST | Gloin781_YST_SP_FW | GCGCGCGAATTCATGAAGAAAATAATTTCATTATTAATT |  |  |
|  | Gloin781_YST_noSP_FW | GCGCGCGAATTCATGAGATTGGATCAAGATGAAG |  |  |
|  | Gloin781_YST_RV | AAGGAAAAAAGCGGCCGCCCAACAAGTTAAAGGTTTAATCC |  |  |
|  | Gloin781_YST_SP_RV | AAGGAAAAAAGCGGCCGCTGATTCAATAACAGATAAAGAAG |  |  |
| GLOIN261 |  |  |  |  |
| Gloin261 CDSΔSP PGGB | Gloin261_MB_F | TTTTGGTCTCAAACAATGGAGGAGGATCTGATAC |  |  |
|  | Gloin261_MB_R | TTTTGGTCTCAAGCCGGTCCTGAAGTAGAACACGTT |  |  |
| Gloin261 CDSΔSP | Gloin261_AttB1 | GGGGACAAGTTTGTACAAAAAAGCAGGCTCAATGGAGGAGGATCTGATAC |  |  |
|  | Gloin261_AttB2 | ggggaccactttgtacaagaaagctgggtaTCAGGTCCTGAAGTAGAACAC |  |  |
| Gloin261 YST | Gloin261_YST_SP_FW | GCGCGCGAATTCATGAACCGTTTACATATCTTAAT |  |  |
|  | Gloin261_YST_noSP_FW | GCGCGCGAATTCATGGAGGAGGATCTGATACCCG |  |  |
|  | Gloin261_YST_RV | AAGGAAAAAAGCGGCCGCGGTCCTGAAGTAGAACACGT |  |  |
|  | Gloin261_YST_SP_RV | AAGGAAAAAAGCGGCCGCTGAAAAAGCGGTAACGAAGA |  |  |
| RiSP749 |  |  |  |  |
| RiSP749 CDSΔSP PGGC | RiSP749_pGGC_START | AGAAGTGAAGCTTGGTCTCAGGCTCCATGAAGAAATACGATCCTCTTCAAG |  |  |
|  | RiSP749_pGGCnoSTOP | AGGGCGAGAATTCGGTCTCACTGACCTGCGATGTTCATTATTTC |  |  |
|  | RiSP749_pGGC_wSTOP | AGGGCGAGAATTCGGTCTCACTGATCACCTGCGATGTTCATTATTTC |  |  |
| RiSP749 CDSΔSP | RiSP749_attb1 | GGGGACAAGTTTGTACAAAAAAGCAGGCTCAATGAAGAAATACGATCCTCTTCAAG |  |  |
|  | RiSP749_attB2_wSTOP | GGGGACCACTTTGTACAAGAAAGCTGGGTTCACCTGCGATGTTCATTATTTC |  |  |
| RiSP749 YST | RiSP749_SP_FW | ATGTGGTATGCGAAAAATTTTCTTC |  |  |
|  | RiSP749_SP_middle | TCTTCAAAATTTAATTTTAATTATAC |  |  |
|  | RiSP749_SP_RV | CGCGTACCACATTTCTTGTATAATT |  |  |
|  | RiSP749_YST_SP_FW | GCGCGCGAATTCATGTGGTATGCGAAAAATTTTCTTC |  |  |
|  | RiSP749_YST_noSP_FW | GCGCGCGAATTCATGAAGAAATACGATCCTCTTCAAG |  |  |
|  | RiSP749_YST_RV | AAGGAAAAAAGCGGCCGCCCTGCGATGTTCATTATTTC |  |  |
|  | RiSP749_YST_SP_RV | AAGGAAAAAAGCGGCCGCCGCGTACCACATTTCTTGTATAATT |  |  |
| Solyc01g073820 (Sl296) |  |  |  |  |
| Sl296 RNAi | Sl296_RNAi_AttB1 | GGGGACAAGTTTGTACAAAAAAGCAGGCTCAAACACGCCTTGAAACTAATTG |  |  |
|  | Sl296_RNAi_AttB2 | ggggaccactttgtacaagaaagctgggtaGAAAACGCGGAGCGTTGAA |  |  |
| Sl296 CDS | Sl296_AttB1 | GGGGACAAGTTTGTACAAAAAAGCAGGCTCAATGGGAAGACAAAACCGG |  |  |
|  | Sl296_AttB2 | ggggaccactttgtacaagaaagctgggtaTTAATAGTAACCGTATCGTC |  |  |
|  | Sl296_AttB3 | GGGGACAACTTTGTATAATAAAGTTGTAATGGGAAGACAAAACCGG |  |  |
| Solyc06g007610.2 (SlGLY) |  |  |  |  |
| SlGLY RNAi | SlGLY_RNAi_AttB1 | GGGGACAAGTTTGTACAAAAAAGCAGGCTCAGCAACTAAGGTTGCTGTCG |  |  |
|  | SlGLY_RNAi_AttB2 | ggggaccactttgtacaagaaagctgggtaCTGATCGGCCTTCCTCTTA |  |  |
| SlGLY CDS | SlGLY_AttB1 | GGGGACAAGTTTGTACAAAAAAGCAGGCTCAATGGCTGAGGAGGCACATA |  |  |
|  | SlGLY_AttB2 | ggggaccactttgtacaagaaagctgggtaTTACTCGGATTCCTTCAC |  |  |
|  | SlGLY_AttB3 | GGGGACAACTTTGTATAATAAAGTTGTAATGGCTGAGGAGGCACATA |  |  |

**Supplementary Table 3.** Oligonucleotide sequences for qRT-PCR analysis.

| qRT-PCR general | |  | |  | |  |
| --- | --- | --- | --- | --- | --- | --- |
| ID | |  | | **SEQUENCE** | |  |
| Solyc05g014470.2 | | Fw | | ACAACTTAACGGCAAATTGACTGG | |  |
|  | | Rv | | TTACCCTCTGATTCCTCCTTGATTG | |  |
| Solyc06g009960 | | Fw | | acaggcgttcaggtaaggaa | |  |
|  | | Rv | | cttgacaacaccgacagcaa | |  |
| U9UB01 (GLOIN707) | | Fw | | CAGATTCTGATACGATTAGACCAAA | |  |
|  | | Rv | | TTCTCATCAACTTTCCAAACGA | |  |
| U9TEU5 (GLOIN781) | | Fw | | ACCTCTCATCATTTCAGCTGGA | |  |
|  | | Rv | | GGTGGACTTGGGAAATATGCA | |  |
| A0A2H5S4X6 (GLOIN261) | | Fw | | GCGGAAGTTACATGGGATGGA | |  |
|  | | Rv | | ACGGTCACCGAATACACCAT | |  |
| RiSP749 | | Fw | | CAAACGAATTTCACCAAGCA | |  |
|  | | Rv | | GCGCCTCTCGTGACTTTTAT | |  |
| Solyc01g073820 | | Fw | | ATGGCAGCTCAACTTCGTGCTC | |  |
|  | | Rv | | CAAGTCCAGACAAGCCTCTCTTCC | |  |
| Solyc06g007610.2 | | Fw | | GATCAGGAGACACCGCTCAT | |  |
|  | | Rv | | TGTACGAGCACCATCACCAT | |  |
| LOC101266050 | | Fw | | caatcagggatctggatggt | |  |
|  | | Rv | | ttcacgagcaaaatgaccag | |  |
| MTR_3g085850 | | Fw | | TGCCTACCGTCGATGTTTCAGT | |  |
|  | | Rv | | TTGCCCTCTGATTCCTCCTTG | |  |
| DQ282611.1 | | Fw | | TTTCGTCCCAATATCCGGCT | |  |
|  | | Rv | | AGTGGAAGACGAAGGGGTTT | |  |
| TURBO | | Fw | | CAGACAAGGTGCGAGTCAAA | |  |
|  | | Rv | | GGCCAGAGTATTCCTGTCCA | |  |
| AT2G37620 | | Fw | | GGCTCCTCTTAACCCAAAGGC | |  |
|  | | Rv | | CACACCATCACCAGAATCCAGC | |  |
| AT1G13320 | | Fw | | TAACGTGGCCAAAATGATGC | |  |
|  | | Rv | | GTTCTCCACAACCGCTTGGT | |  |
| AT5G62690 | | Fw | | GAGCCTTACAACGCTACTCTGTCTGTC | |  |
|  | | Rv | | CACCAGACATAGTAGCAGAAATCAAG | |  |
| qRT-PCR_RiSP749 DEGs | | | | | | |
| ID SEQUENCE | | | | | | |
| Solyc06g034000 |  | Fw |  | TGCCCAAATTTGCAGGGCTATC | |  |
|  |  | Rv |  | GGTCTCAAGTAGTTGATCCATCGG | |  |
| Solyc07g054770 |  | Fw |  | ATCTACGCTCGTATTCACAAGCC | |  |
|  |  | Rv |  | AAGACTCCTCAGACTGCTTGGC | |  |
| Solyc01g108540 |  | Fw |  | GCTTCACATGCTAATGGTGATGGC | |  |
|  |  | Rv |  | ACCCGACTGAAATCGGCATGTTC | |  |
| Solyc01g018020 |  | Fw |  | TGGGACAAGGTGTTGCGAATGC | |  |
|  |  | Rv |  | AGCCATCACCCATGATGCAATACG | |  |
| Solyc01g111630 |  | Fw |  | TACATGAAACCCGGCCTTGCAG | |  |
|  |  | Rv |  | ATTCCCTCGCGAGTCCACTTAG | |  |
| Solyc12g088760 |  | Fw |  | CCATGATGACCACAGCCGATTC | |  |
|  |  | Rv |  | TGTCTCTACCGATGTCTCGGATGG | |  |
| Solyc01g107800 |  | Fw |  | TCCGCCGTTGTAAAGCTCCTTG | |  |
|  |  | Rv |  | TGCCACAACAGGGTTTCGTGAG | |  |
| Solyc04g078250 |  | Fw  Rv |  | TGAGGTCAACGGACCACTGTTTG  TGAAGGCAACATAACCTGCAGTCC | |  |
| Solyc12g042360 |  | Fw  Rv |  | AATGCCTCGTCAAAGGTGTGG  CATGTTGCATCCTGTCGATGTTG | |  |
| Solyc07g063930 |  | Fw |  | TGGAGCTGCAAGTTCTACGGTATC | |  |
|  |  | Rv |  | TGTCCAAATACTCCAGCACCAATC | |  |
| Solyc08g074690 |  | Fw |  | AGATGAAGGTGTGAAGGCGAAGG | |  |
|  |  | Rv |  | AGGCAAATTCGCAAAGCTACCG | |  |
| Solyc03g093560 |  | Fw |  | AACGTAGCAATACCGGCGAAGC | |  |
|  |  | Rv |  | GTCACGAATCTCCGCTGCAAAC | |  |
| Solyc03g113370 |  | Fw  Rv |  | TGCACTTGCTGTATCTTCTGCAC  AGAGGCGATGATGACTGAACACC | |  |
| qRT-PCR_GLOIN781 DEGs | | | | | | |
| Solyc00g071180 | | Fw |  | TTGGGTGAAGGAATGGGAGGAC |  | |
|  | | Rv |  | GGTTTGTTTGGGAATGGAACATCG |  | |
| Solyc01g091010 | | Fw |  | AAAGCCACCAGTCGCAAACAGACC |  | |
|  | | Rv |  | TTCTTTGCAGCAGCACTGAAGGC |  | |
| Solyc01g098800 | | Fw |  | AACTGCCTGCTGCCTTTCTTGG |  | |
|  | | Rv |  | AGCCACGTCTGCAACAAGATCAC |  | |
| Solyc02g088560 | | Fw |  | TCCGCTTCTTCCTCTCAAAGTGG |  | |
|  | | Rv |  | TGTCGTCGCTGTTATCAGGTTG |  | |
| Solyc04g009490 | | Fw |  | TCCCATCTCCTTCAACTCCCTTGG |  | |
|  | | Rv |  | GTGATGCAACGCCAGCACTAAC |  | |
| Solyc04g081130 | | Fw |  | CAGCTGGATGGCAAATTGCAAGG |  | |
|  | | Rv |  | TACCGTCTCGGAGTAGAACTGCTG |  | |
| Solyc06g061100 | | Fw |  | GGCTGAATAGAGGACTAGGGACAC |  | |
|  | | Rv |  | CATGTATGTAGTCACCGCTCCAAC |  | |
| Solyc06g066560 | | Fw |  | TGTTACCGGAGGATTGACTGTTCC |  | |
|  | | Rv |  | ACTCCTTCAGTAGCTCCTACACC |  | |
| Solyc08g080030 | | Fw |  | TTTGTTTGTGCTGGCTCTTGGC |  | |
|  | | Rv |  | TCGTTTCCCACCAACCTTGGAAC |  | |
| Solyc09g005260 | | Fw |  | TGGGAGTTGCCTTAGGTTCAGC |  | |
|  | | Rv |  | ACAATCACACACAAGGGAACCAC |  | |
| Solyc10g050690 | | Fw |  | CCAGGTCTAGCACTTGGTGTTG |  | |
|  | | Rv |  | GCGCAAACTCTCTGTGTCGATG |  | |
| Solyc10g050730 | | Fw |  | GGTTGTGGAGAGCATAAAGGCATC |  | |
|  | | Rv |  | GGCATCACAGCAAACTTCATGTTC |  | |
| Solyc09g092590 | | Fw |  | CCAGGTCTAGCACTTGGTGTTG |  | |
|  | | Rv |  | GCGCAAACTCTCTGTGTCGATG |  | |
| Solyc09g092600 | | Fw |  | AGTTGCTTCCATTTGGTGCAGGAC |  | |
|  | | Rv |  | TCGCAACACCAAGTGCAATACCTG |  | |
| Solyc11g050940 | | Fw |  | CTGACTGTTGCACCCGGTATTC |  | |
|  | | Rv |  | AGCTCTCCTACATACCTCTTGCTC |  | |
| Solyc12g008900 | | Fw |  | CCCTAGAGTATGGCTTAGCACCTG |  | |
|  | | Rv |  | GAGAGTGTACCACCAACGGTAAGG |  | |
| Solyc12g056810 | | Fw |  | GGTCCATCACACATGACCCTAACG |  | |
|  | | Rv |  | ACCTCTCTGGCTTAAACGTGGAC |  | |
| Solyc12g096890 | | Fw |  | TCTCATGTGGACTCAAGATTCGC |  | |
|  | | Rv |  | GCCTCGAATATCAAGCCAACAAAC |  | |
| Solyc01g107390 | | Fw |  | AACCGGAGATTCAACGGATCGC |  | |
|  | | Rv |  | AGCTGACGTTCCAGAGCTAGTG |  | |
| Solyc01g090810 | | Fw |  | AGTGGTGCAGGAAGTGATGGTG |  | |
|  | | Rv |  | AGCAGCGGCTATTAGAGACGAG |  | |
| Solyc05g052600 | | Fw |  | ACAATCCTGATTACGCCAAGCTG |  | |
|  | | Rv |  | TCCTCCGGTGTATCTCAAGGTG |  | |
| Solyc07g008020 | | Fw |  | GCGGCGCGATGAATTATGTCAC |  | |
|  | | Rv |  | TTGATCCTCTGCCACCACTAACG |  | |
| qRT-PCR_GLOIN707 DEGs | | | | | | |
| Solyc02g032910 | | Fw |  | TTGGTGTAGGGCCGGTTATTGG |  | |
|  | | Rv |  | AATCCGCCACCGTTGTTTGGAC |  | |
| Solyc02g077970 | | Fw |  | TCGAAGACGATTCCTCGAACAAC |  | |
|  | | Rv |  | TCCGATGCATGGATTCCCTCAG |  | |
| Solyc03g026050 | | Fw |  | AACATCTCCACTGGATTGTGACG |  | |
|  | | Rv |  | TCGTAGCACACTATCTCCCTTCC |  | |
| Solyc03g114080 | | Fw |  | CCCTTGTTGCGTTCTACAGTTCC |  | |
|  | | Rv |  | ACTGCCCTGAGATGAATGGAGATG |  | |
| Solyc05g050880 | | Fw |  | GACATTCCTGCACCAACTTTGAAC |  | |
|  | | Rv |  | TCCTATCGTGTGAGACCCTGAG |  | |
| Solyc06g054060 | | Fw |  | ATGGAGGTGGTTCGTCGTTAGG |  | |
|  | | Rv |  | AGGTTGCATGCAGCAGGTAATG |  | |
| Solyc06g065620 | | Fw |  | CTCCCGCTCGTTCGAATATCAG |  | |
|  | | Rv |  | TCCGGTTTGGTTGAGACAACAG |  | |
| Solyc11g020990 | | Fw |  | GGCGGATAATAATCACGGGAAGCC |  | |
|  | | Rv |  | GCATATGCGATTCGAGGGTCAC |  | |
| Solyc11g032060 | | Fw |  | ATCGGTGTGCTTCTGGGATTGC |  | |
|  | | Rv |  | CGCATTGTATCCACGTTGAACTCC |  | |
| Solyc10g084560 | | Fw |  | CTTCAGCTGCAGTCATTGGTGTC |  | |
|  | | Rv |  | GAAATGTGAGGAGCAGCCATCG |  | |
| Solyc01g099880 | | Fw |  | GCAGCTACGAGGACAGTGATAAAG |  | |
|  | | Rv |  | AGCCTATCCCATTTGGCACTCC |  | |
| Solyc01g101050 | | Fw |  | CGCTATGGAACAGTCCTTCAACGG |  | |
|  | | Rv |  | CCCAATTCGCACCATGAATCGC |  | |
| Solyc01g102380 | | Fw |  | TTGCGTTGCTGATCTCACCTCAAC |  | |
|  | | Rv |  | AGAGAAATCAGCCGCGGAGAAC |  | |
| Solyc04g074050 | | Fw |  | TGAAATAGGCCATTTGCCAGAGC |  | |
|  | | Rv |  | CTTCTTGGGATTGTCCCTGTAAGC |  | |
| Solyc04g082120 | | Fw |  | CCCTTCCATCATGCTATTGACTGC |  | |
|  | | Rv |  | CACAGGACGTATTGCATGGTAGC |  | |
| Solyc09g066410 | | Fw |  | GCCACCACATTTGTCGTGTCTG |  | |
|  | | Rv |  | GCAGCTGATATTCCATGGCAAGTG |  | |
| Solyc06g035710 | | Fw |  | AGTGGGAATGCCTGTAGCTGTTG |  | |
|  | | Rv |  | GGCCAACAATCCCAACCATAGACC |  | |
| Solyc09g015440 | | Fw |  | GCTTGCATCCAAGATCTCACAACG |  | |
|  | | Rv |  | GGTTGGAGCAACGCGATTTGTG |  | |
| Solyc12g009920 | | Fw |  | ATTCTTGGGACATTCATCGACAGG |  | |
|  | | Rv |  | ATCGAAACTGATTGGCCTAGTAGC |  | |
| Solyc12g044190 | | Fw |  | CCCAACTTGGAAGAGCTAACCATC |  | |
|  | | Rv |  | GTTGGTCAGAGCATAGAGTTACGC |  | |
| Solyc08g080590 | | Fw |  | GCCAAACGTGGACGATAAACGC |  | |
|  | | Rv |  | GCATCGAAATTGCATCCGGTACG |  | |

**Supplementary Table 4.** Relative expression of effectors normalized against the fungal housekeeping gene *RiEF1α.* No statistical differences were found between not-enriched and enriched samples per week tested with Student’s *t*-test, P > 0.05.

|  |  | *GLOIN707* | *GLOIN781* | *GLOIN261* | *RiSP749* |
| --- | --- | --- | --- | --- | --- |
| Week 2 | not-enriched sample 3 | 0,00 | 0,00 | 0,01 | 1,95 |
|  | not-enriched sample 2 | 0,07 | 0,07 | 1,00 | 0,31 |
|  | not-enriched sample 1 | 1,00 | 1,00 | 1,00 | 1,00 |
|  | SlPT4-enriched sample 3 | 0,09 | 0,25 | 1,00 | 0,03 |
|  | SlPT4-enriched sample 2 | 0,14 | 0,13 | 0,04 | 1,22 |
|  | SlPT4-enriched sample 1 | 0,99 | 0,84 | 1,00 | 1,29 |
| Week 4 | not-enriched sample 3 | 4,70 | 1513,14 | 3,23 | 0,01 |
|  | not-enriched sample 2 | 379,16 | 62576,50 | 16,87 | 0,00 |
|  | not-enriched sample 1 | 1,00 | 1,00 | 1,00 | 1,00 |
|  | SlPT4-enriched sample 3 | 0,05 | 15,71 | 8,63 | 0,00 |
|  | SlPT4-enriched sample 2 | 0,08 | 15,67 | 8,27 | 0,00 |
|  | SlPT4-enriched sample 1 | 0,13 | 48,95 | 10,17 | 0,00 |
| Week 6 | not-enriched sample 3 | 0,65 | 0,05 | 1,62 | 0,32 |
|  | not-enriched sample 2 | 1,00 | 1,00 | 1,00 | 1,00 |
|  | not-enriched sample 1 | 16,32 | 0,97 | 1,91 | 1,35 |
|  | SlPT4-enriched sample 3 | 1,10 | 0,07 | 1,82 | 0,33 |
|  | SlPT4-enriched sample 2 | 0,76 | 0,05 | 2,84 | 0,33 |
|  | SlPT4-enriched sample 1 | 0,83 | 0,05 | 2,89 | 0,24 |

**Supplementary Table 5.** Differentially expressed genes 24 h after *GLOIN707, GLOIN781* and *RiSP749* induction.

*See excel file*
